# Supplementary material for: Wing Musculature Reconstruction in Extinct Flightless Auks (Pinguinus and Mancalla) Reveals Incomplete Convergence with Penguins (Spheniscidae) Due to Differing Ancestral States
Source: Integr Org Biol. 2020 Nov 11;3(1):obaa040. doi: 10.1093/iob/obaa040 (PMC8271220; doi:10.1093/iob/obaa040)
Supplement: obaa040_Supplementary_Data [file obaa040_supplementary_data.pdf]

Supplementary Figures S1–S32

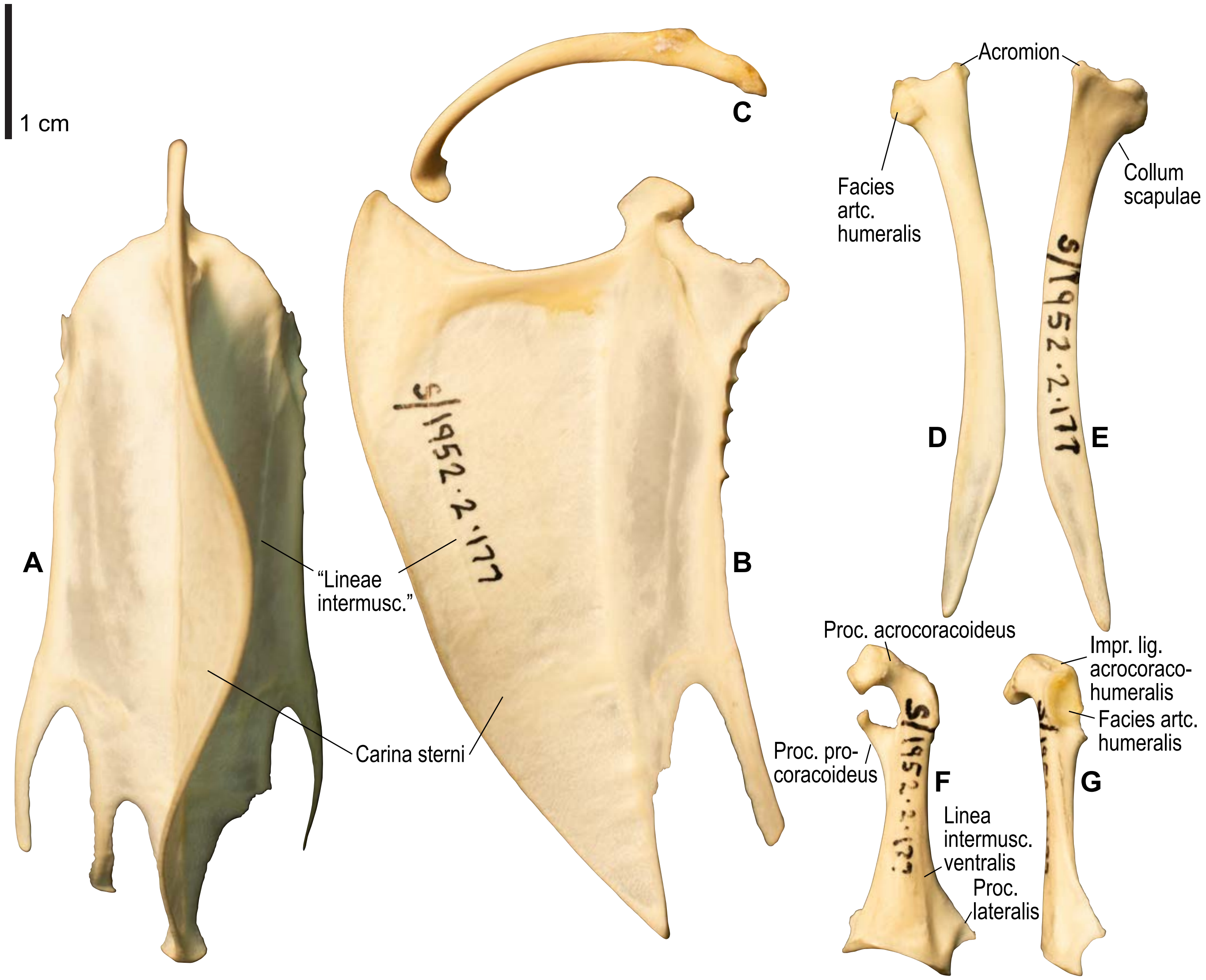

Figure S1. Osteology of the pectoral girdle of *Pluvialis apricaria*. Drawn on NHMUK S/1952.2.178 (furcula) and S/1952.2.177 (other elements). Sternum in ventral (**A**) and left lateral (**B**) views; furcula in left lateral view (**C**); left scapula in lateral (**D**) and medial (**E**) views; left coracoid in ventral (**F**) and lateral (**G**) views. **B** and **C** are roughly aligned in their original relative positions and orientations. Major osteological landmarks mentioned in text are designated. Abbreviations: artc., articularis; impr., impressio; intermusc., intermuscularis/intermusculares; lig., ligamenti; m., musculi; proc., processus; tuberc., tuberculum.

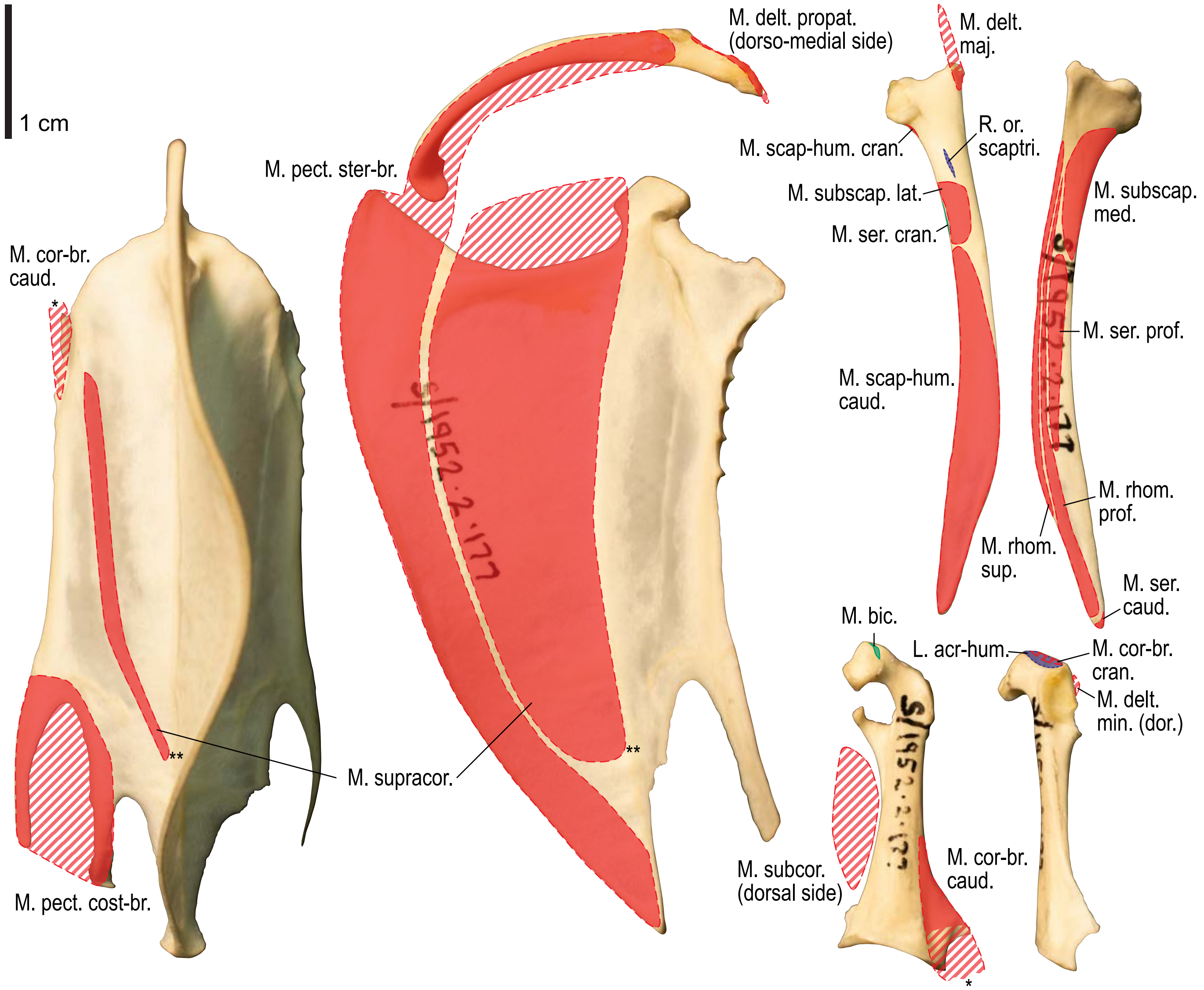

Figure S2. Osteological correlates of major wing muscles and ligaments in the pectoral girdle of *Pluvialis apricaria*. Drawn on NHMUK S/1952.2.178 (furcula) and S/1952.2.177 (other elements). Note that only reliably identified attachment sites are shown, and the gaps between some adjacent attachment sites are exaggerated for distinction. Asterisks denote continuous attachment sites across panels. Red fill with broken outline, fleshy (direct) attachment of muscles; green fill with solid outline, tendinous/aponeurotic (indirect) attachment of muscles; blue fill with dotted outline, attachment of ligaments; stroked fill, attachment on ligaments/membranes.

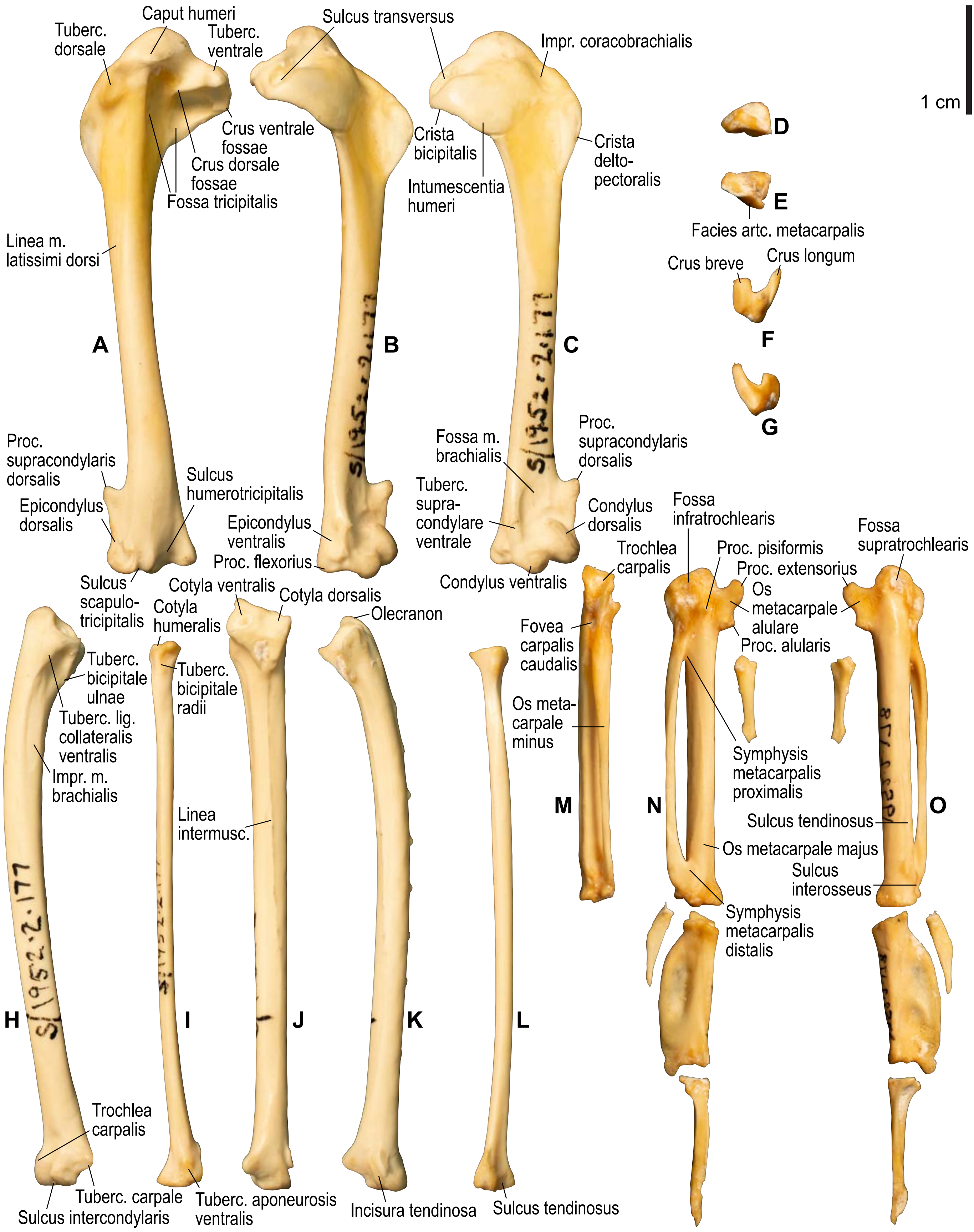

Figure S3. Osteology of the wing of *Pluvialis apricaria*. Drawn on NHMUK S/1952.2.178 (manual elements) and S/1952.2.177 (other elements). Left humerus in caudal (**A**), ventral (**B**), and cranial (**C**) views; left radiale in cranial (**D**) and caudal (**E**) views; left ulnare in proximal (**F**) and distal (**G**) views; left ulna in ventral (**H**), cranial (**J**), and dorsal (**K**) views; left radius in ventral (**I**) and cranial (**L**) views; right carpometacarpus and phalanges (mirrored for comparison) in caudal (**M**; phalanges not shown), ventral (**N**), and dorsal (**O**) views. See Figure S1 for abbreviations.

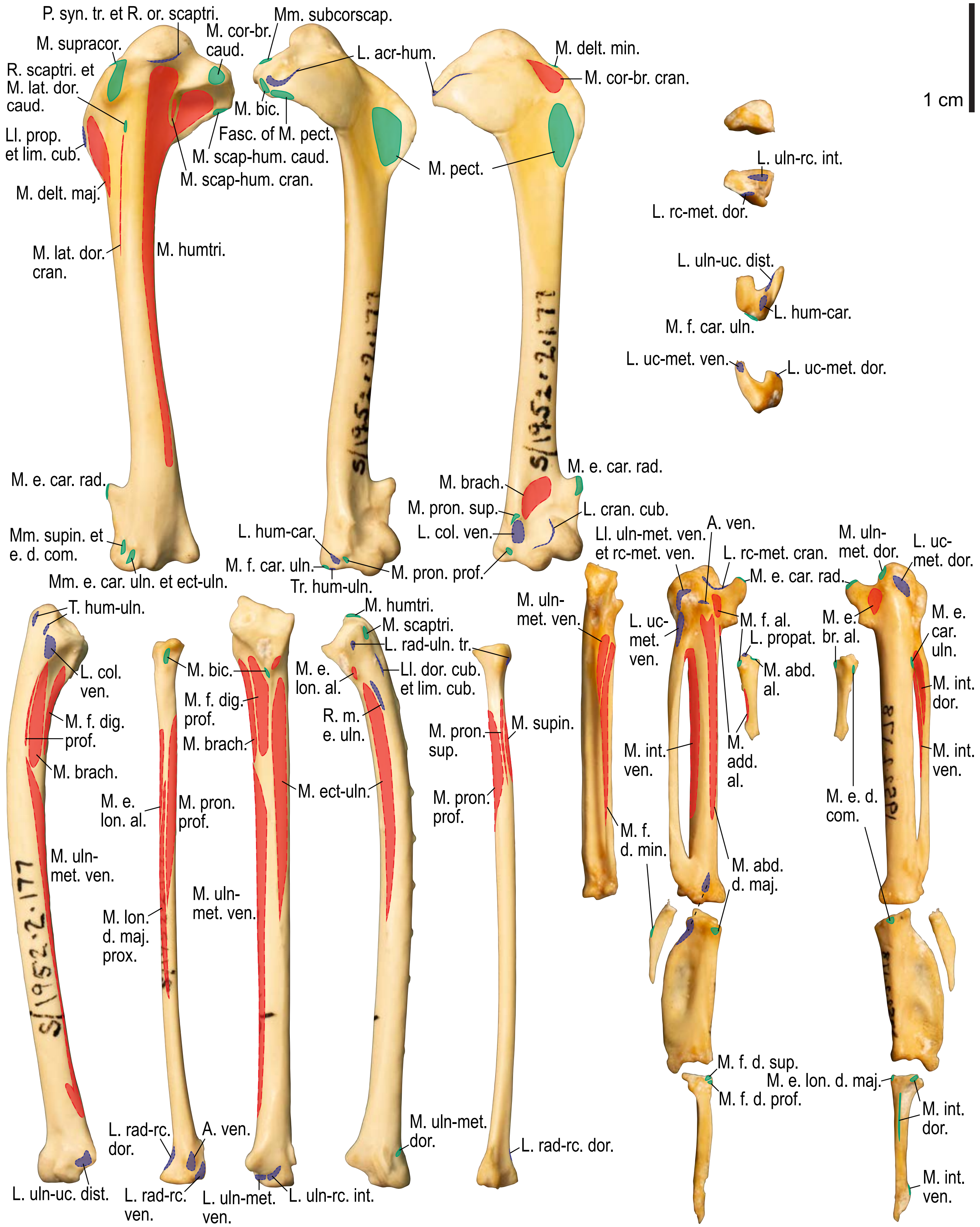

Figure S4. Osteological correlates of major wing muscles and ligaments in the wing of *Pluvialis apricaria*. Drawn on NHMUK S/1952.2.178 (manual elements) and S/1952.2.177 (other elements). Manual elements are mirrored for comparison. Due to space restrictions, the labels for some distal wing ligaments are not shown; broken lines show correspondence of attachment sites for these ligaments. See Figure S2 for legends.

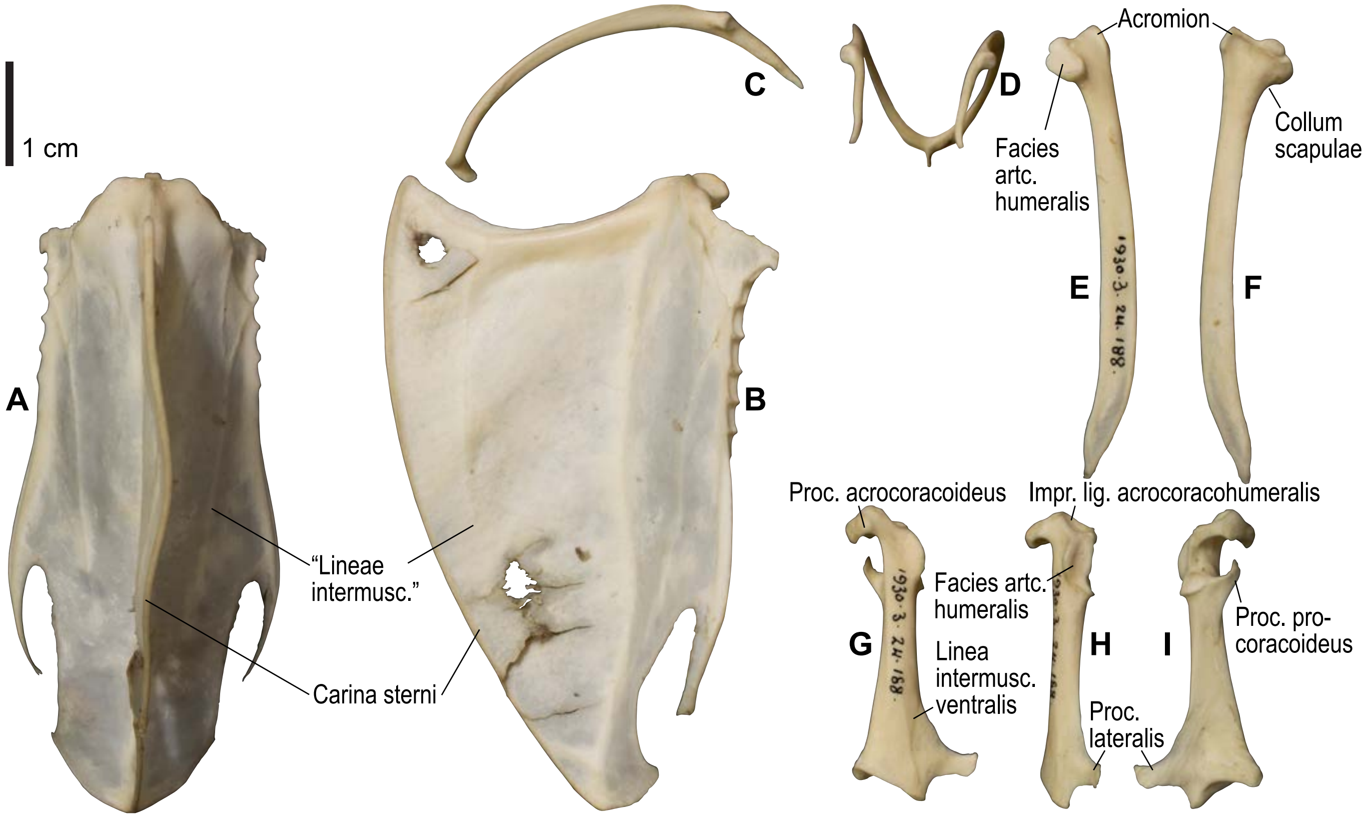

Figure S5. Osteology of the pectoral girdle of *Scolopax rusticola*. Drawn on NHMUK 1930.3-24.188. Sternum in ventral (**A**) and left lateral (**B**) views; furcula in left lateral (**C**) and dorsal (**D**) views; left scapula in lateral (**E**) and medial (**F**) views; left coracoid in ventral (**G**), lateral (**H**), and dorsal (**I**) views. **B** and **C** are roughly aligned in their original relative positions and orientations. See Figure S1 for abbreviations.

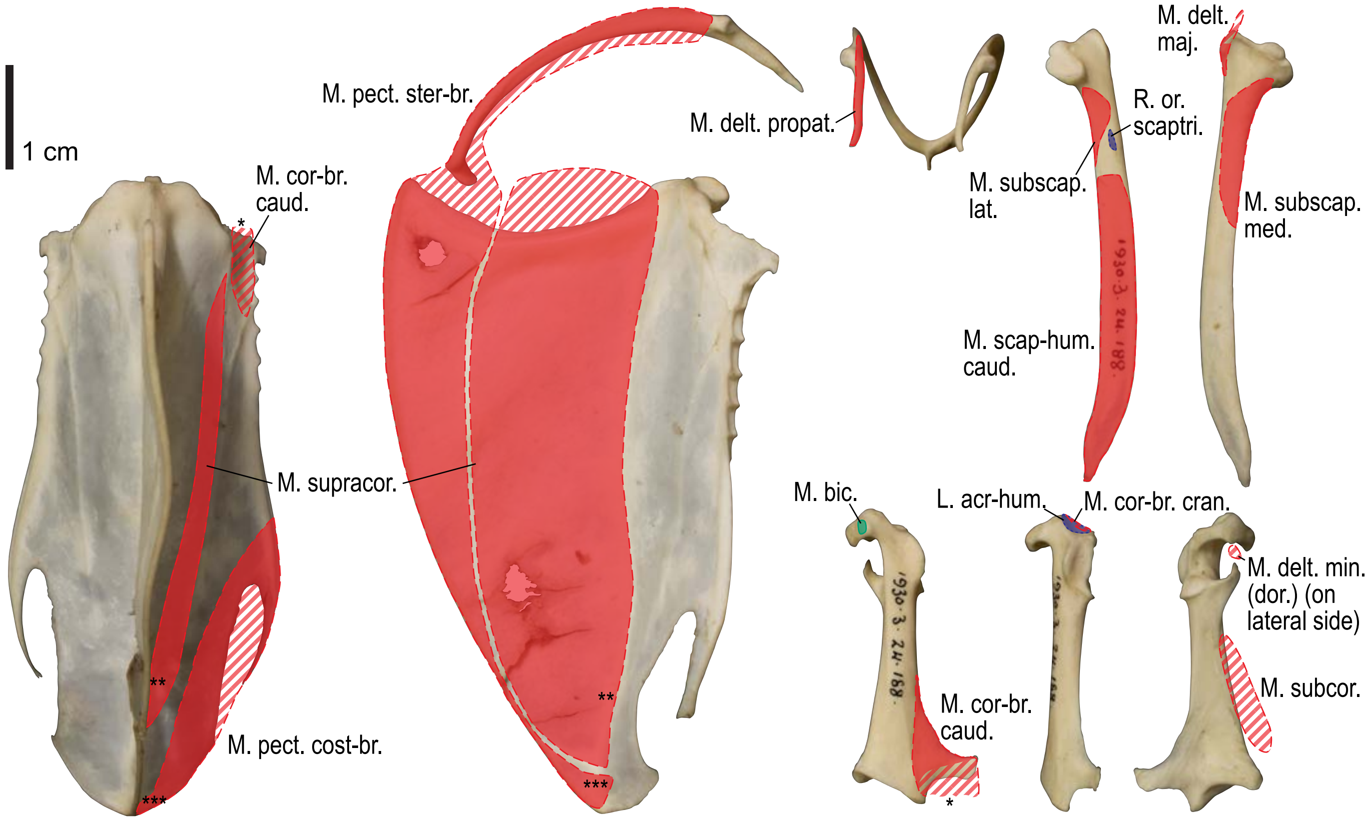

Figure S6. Osteological correlates of major wing muscles and ligaments in the pectoral girdle of *Scolopax rusticola*. Drawn on NHMUK 1930.3-24.188. See Figure S2 for legends.

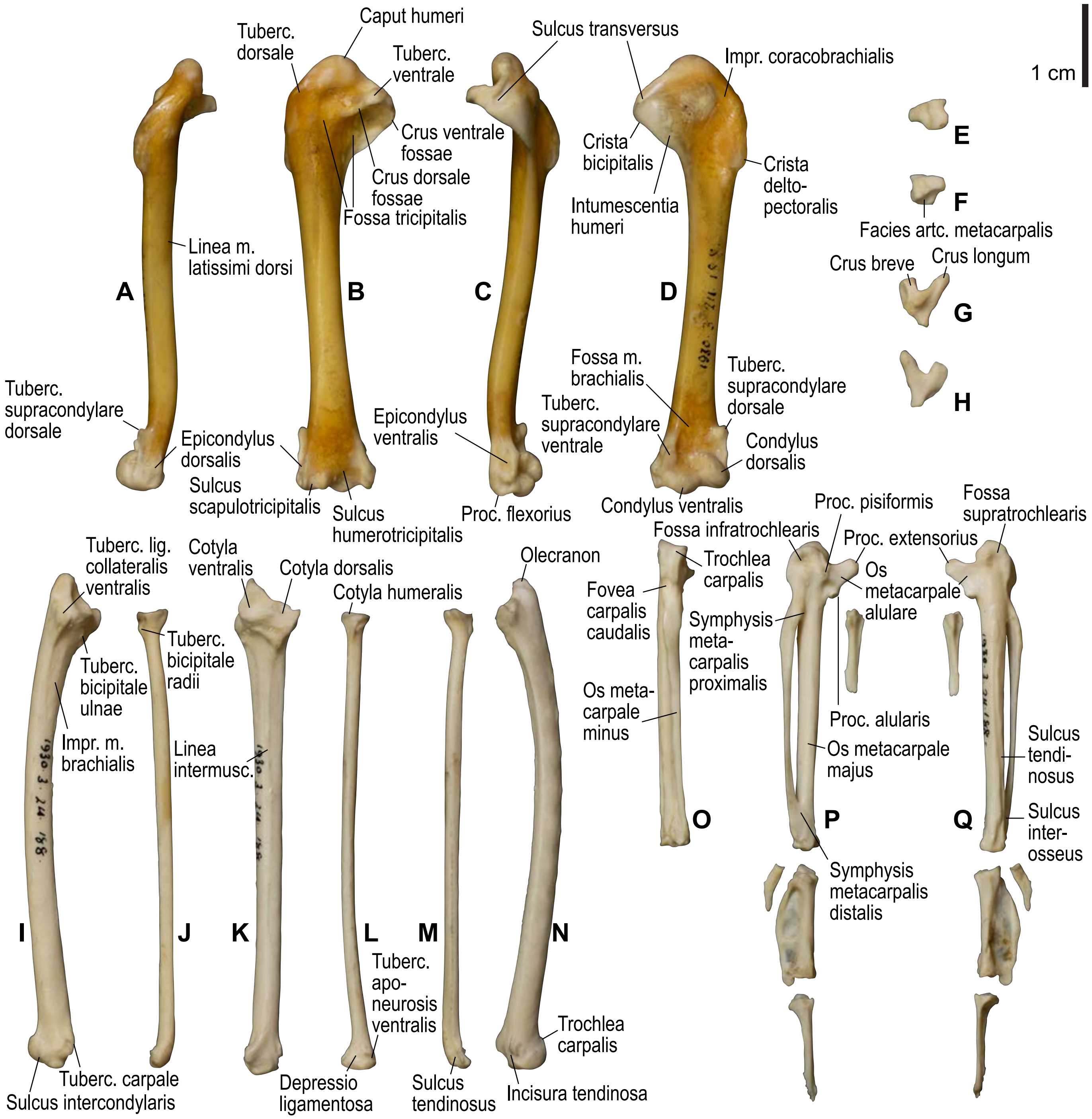

Figure S7. Osteology of the wing of *Scolopax rusticola*. Drawn on NHMUK 1930.3-24.188. Left humerus in dorsal (**A**), caudal (**B**), ventral (**C**), and cranial (**D**) views; left radiale in cranial (**E**) and caudal (**F**) views; left ulnare in proximal (**G**) and distal (**H**) views; left ulna in ventral (**I**), cranial (**K**), and dorsal (**N**) views; left radius in ventral (**J**), caudal (**L**), and dorsal (**L**) views; left carpometacarpus and phalanges in caudal (**O**; phalanges not shown), ventral (**P**), and dorsal (**Q**) views. See Figure S1 for abbreviations.

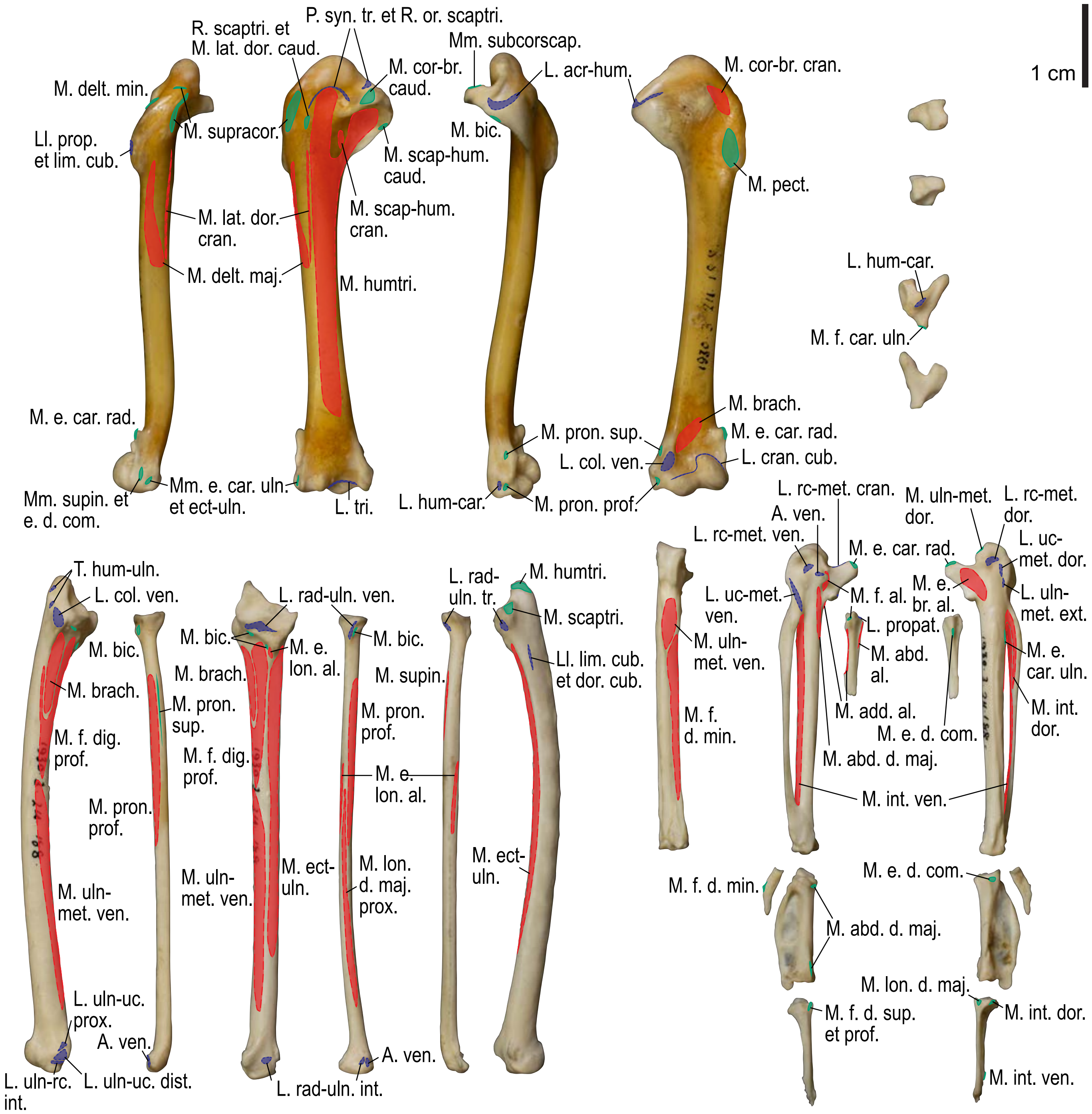

Figure S8. Osteological correlates of major wing muscles and ligaments in the wing of *Scolopax rusticola*. Drawn on NHMUK 1930.3-24.188. See Figure S2 for legends.

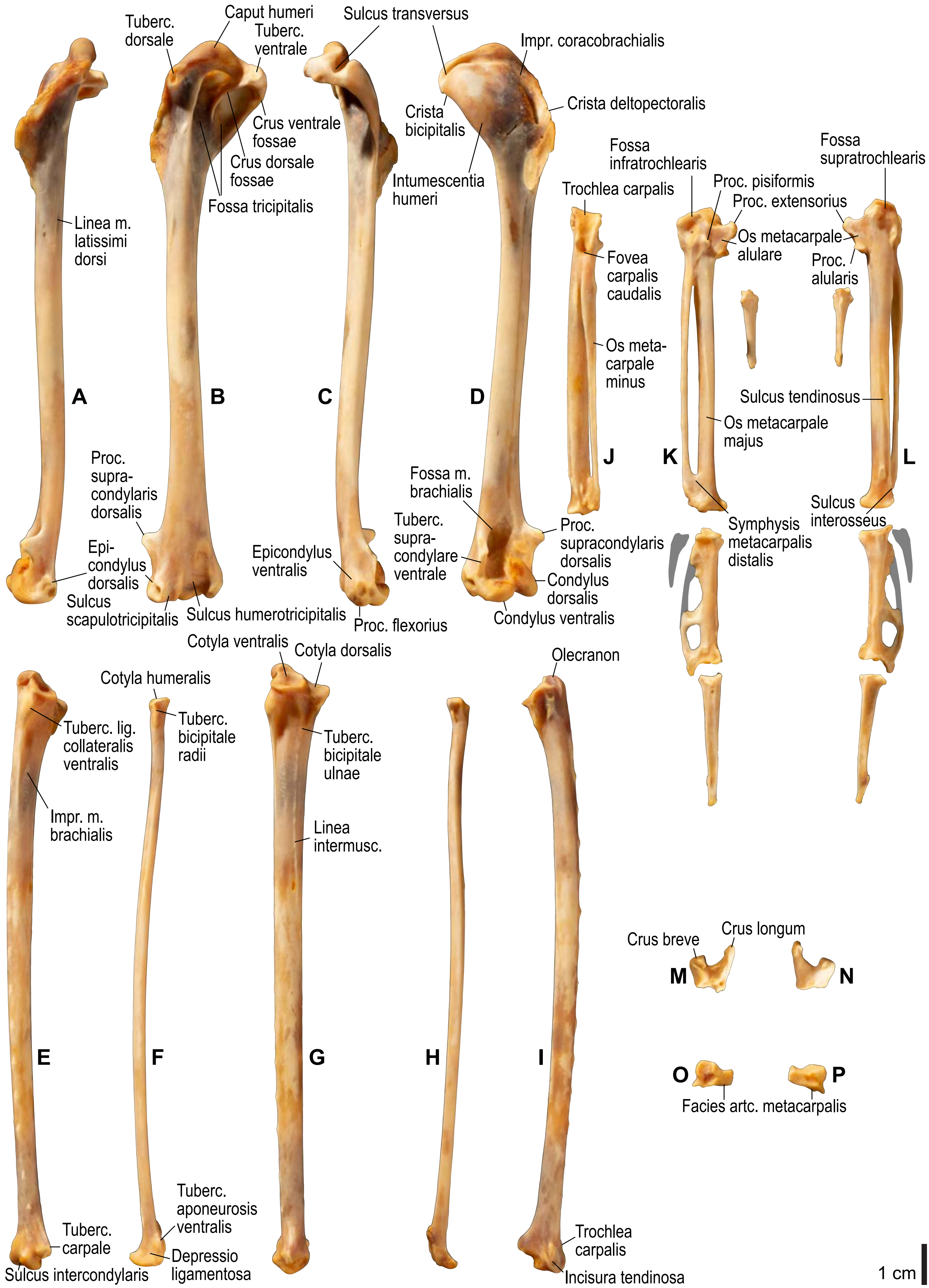

Figure S9. Osteology of the wing of *Larus schistisagus*. Drawn on KUGM RAJ AO13071502. Left humerus in dorsal (**A**), caudal (**B**), ventral (**C**), and cranial (**D**) views; left ulna in ventral (**E**), cranial (**G**), and dorsal (**I**) views; left radius in ventral (**F**) and dorsal (**H**) views; left carpometacarpus and phalanges in caudal (**J**; phalanges not shown), ventral (**K**), and dorsal (**L**) views; left ulnare in proximal (**M**) and distal (**N**) views; left radiale in cranial (**O**) and caudal (**P**) views. Approximate outlines of missing/broken phalanges are shown with gray shading. See Figure S1 for abbreviations.

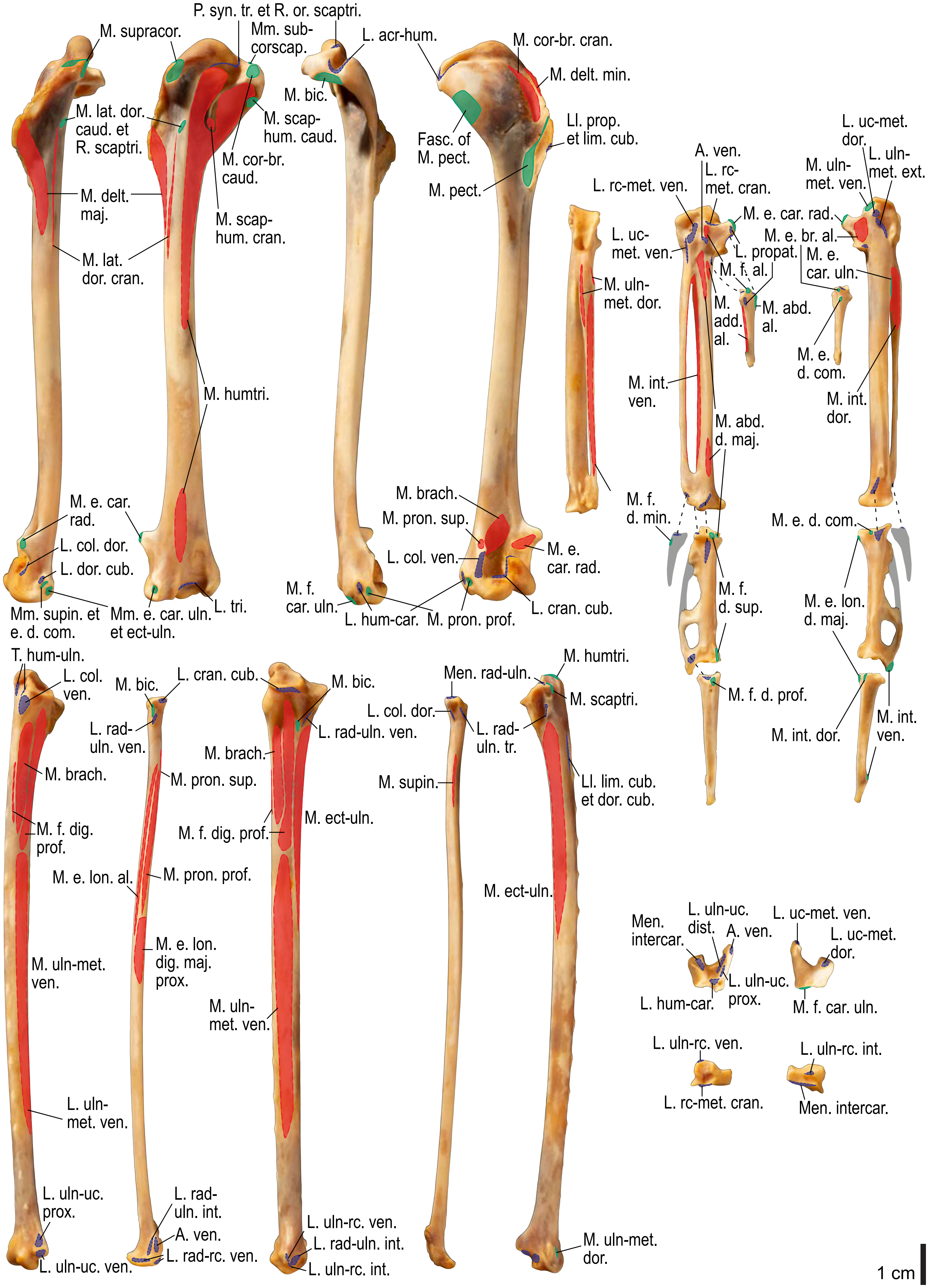

Figure S10. Osteological correlates of major wing muscles and ligaments in the wing of *Larus schistisagus*. Drawn on KUGM RAJ AO13071502. See Figures S2, S4, and S9 for legends.

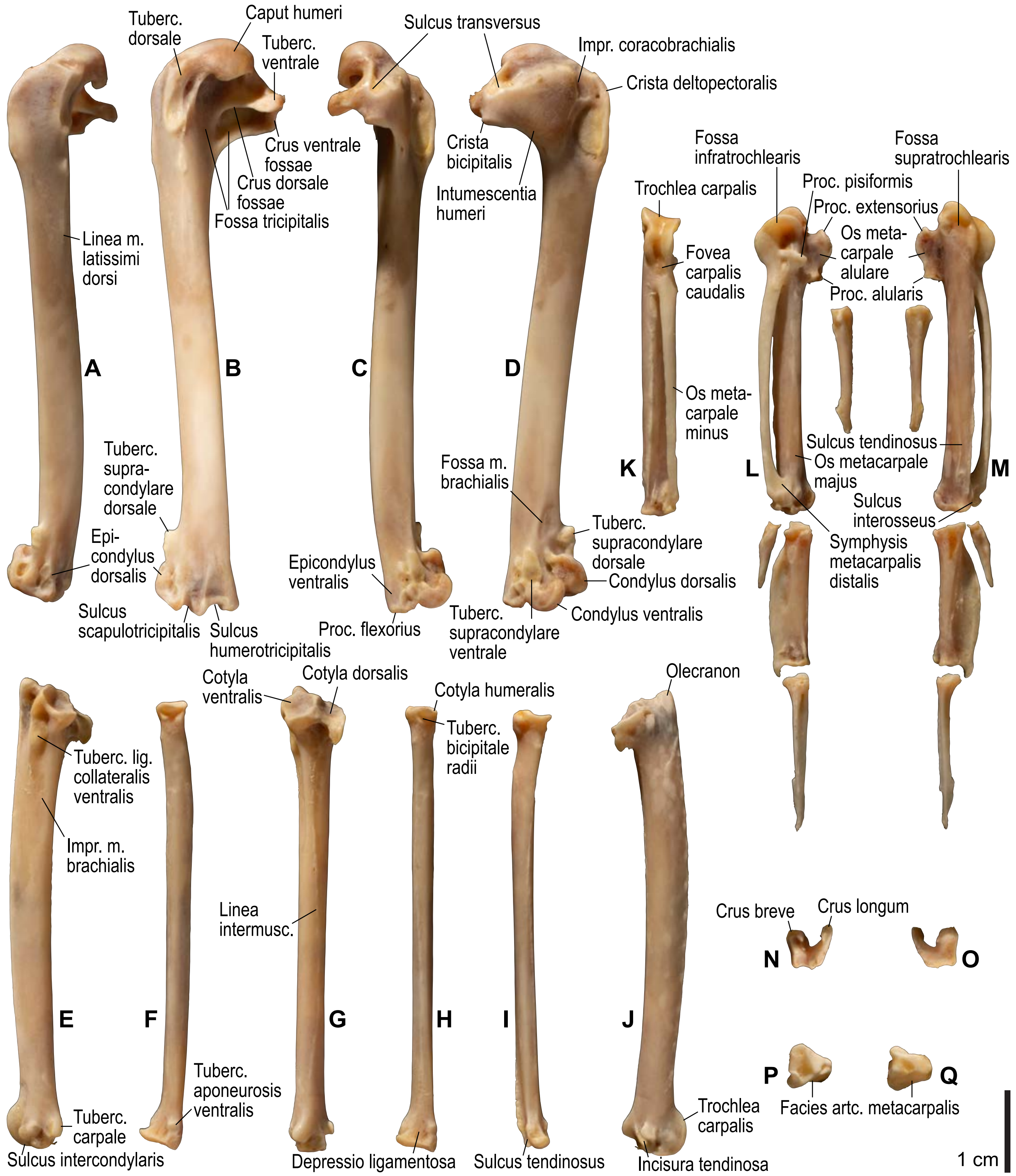

Figure S11. Osteology of the wing of *Cerorhinca monocerata*. Drawn on KUGM RAJ AO13070802. Left humerus in dorsal (**A**), caudal (**B**), ventral (**C**), and cranial (**D**) views; left ulna in ventral (**E**), cranial (**G**), and dorsal (**J**) views; left radius in ventral (**F**), caudal (**H**), and dorsocaudal (**I**) views; left carpometacarpus and phalanges in caudal (**K**; phalanges not shown), ventral (**L**), and dorsal (**M**) views; left ulnare in proximal (**N**) and distal (**O**) views; left radiale in cranial (**P**) and caudal (**Q**) views. See Figure S1 for abbreviations.

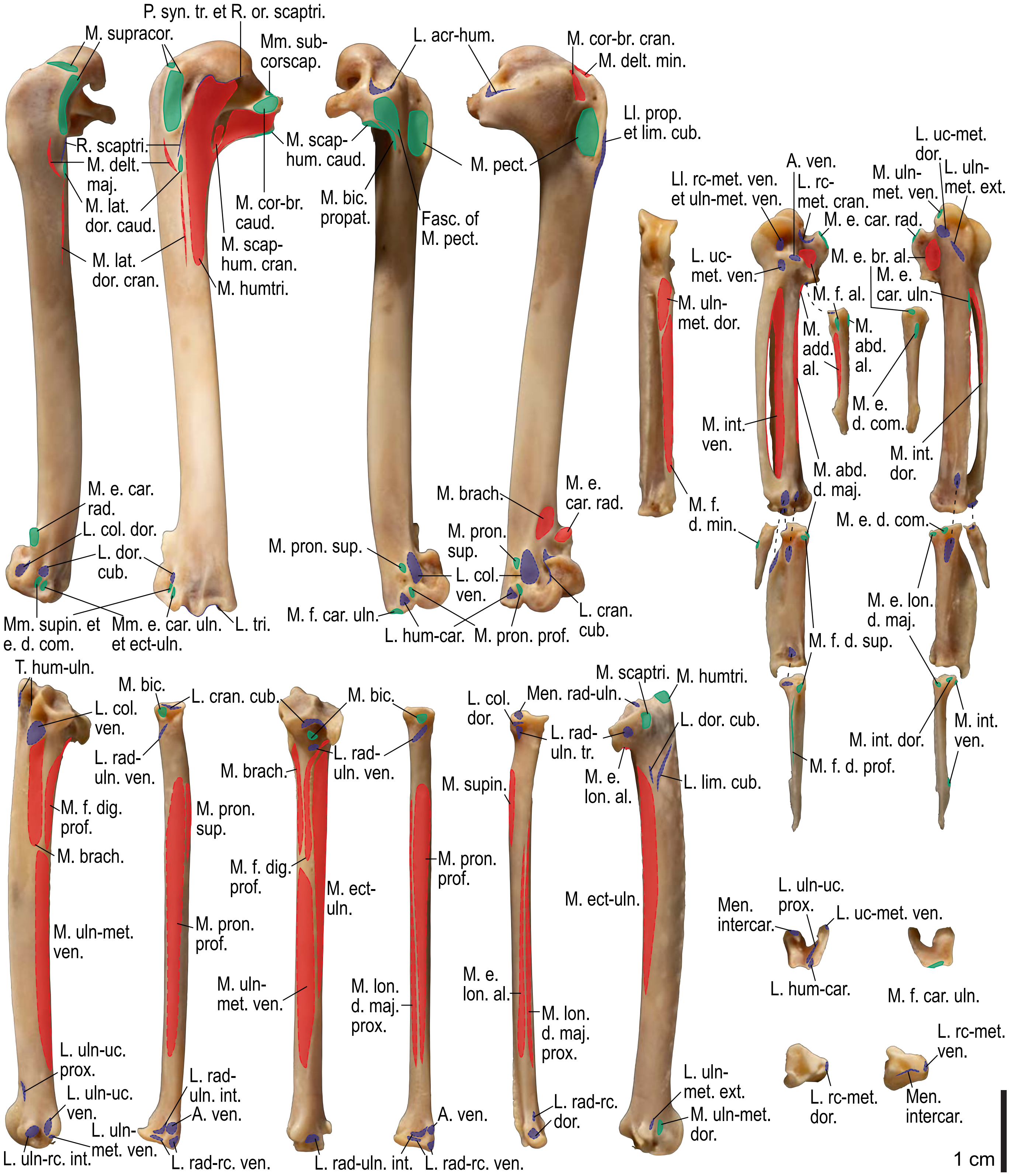

Figure S12. Osteological correlates of major wing muscles and ligaments in the wing of *Cerorhinca monocerata*. Drawn on KUGM RAJ AO13070802. See Figures S1 and S2 for legends.

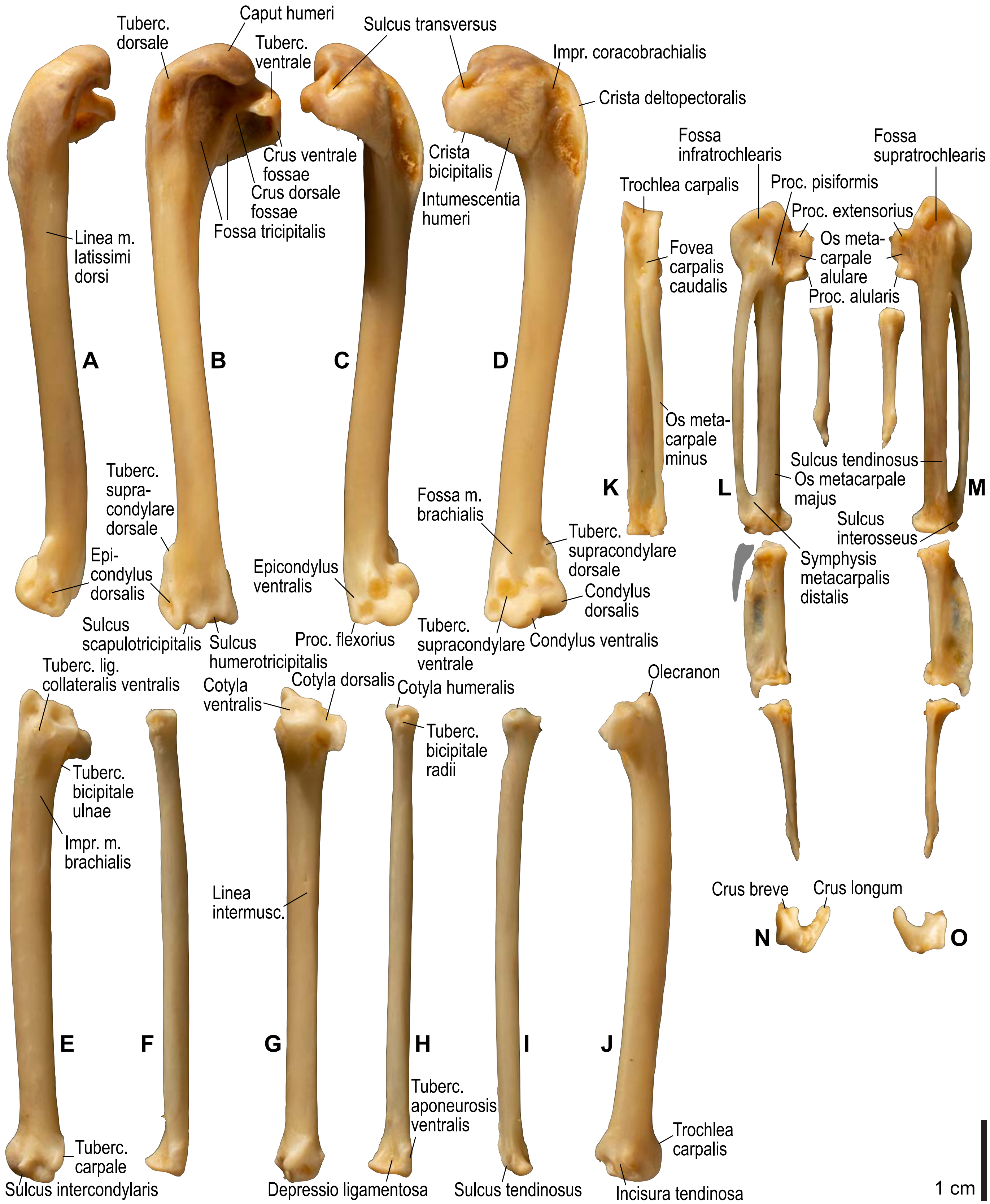

Figure S13. Osteology of the wing of *Cepphus carbo*. Drawn on KUGM RAJ AO13062101. Left humerus in dorsal (**A**), caudal (**B**), ventral (**C**), and cranial (**D**) views; left ulna in ventral (**E**), cranial (**G**), and dorsal (**J**) views; left radius in ventral (**F**), caudal (**H**), and dorsocaudal (**I**) views; left carpometacarpus and phalanges in caudal (**K**; phalanges not shown), ventral (**L**), and dorsal (**M**) views; left ulnare in proximal (**N**) and distal (**O**) views. Approximate outline of the missing phalanx of the minor digit is shown with gray shading. See Figure S1 for abbreviations.

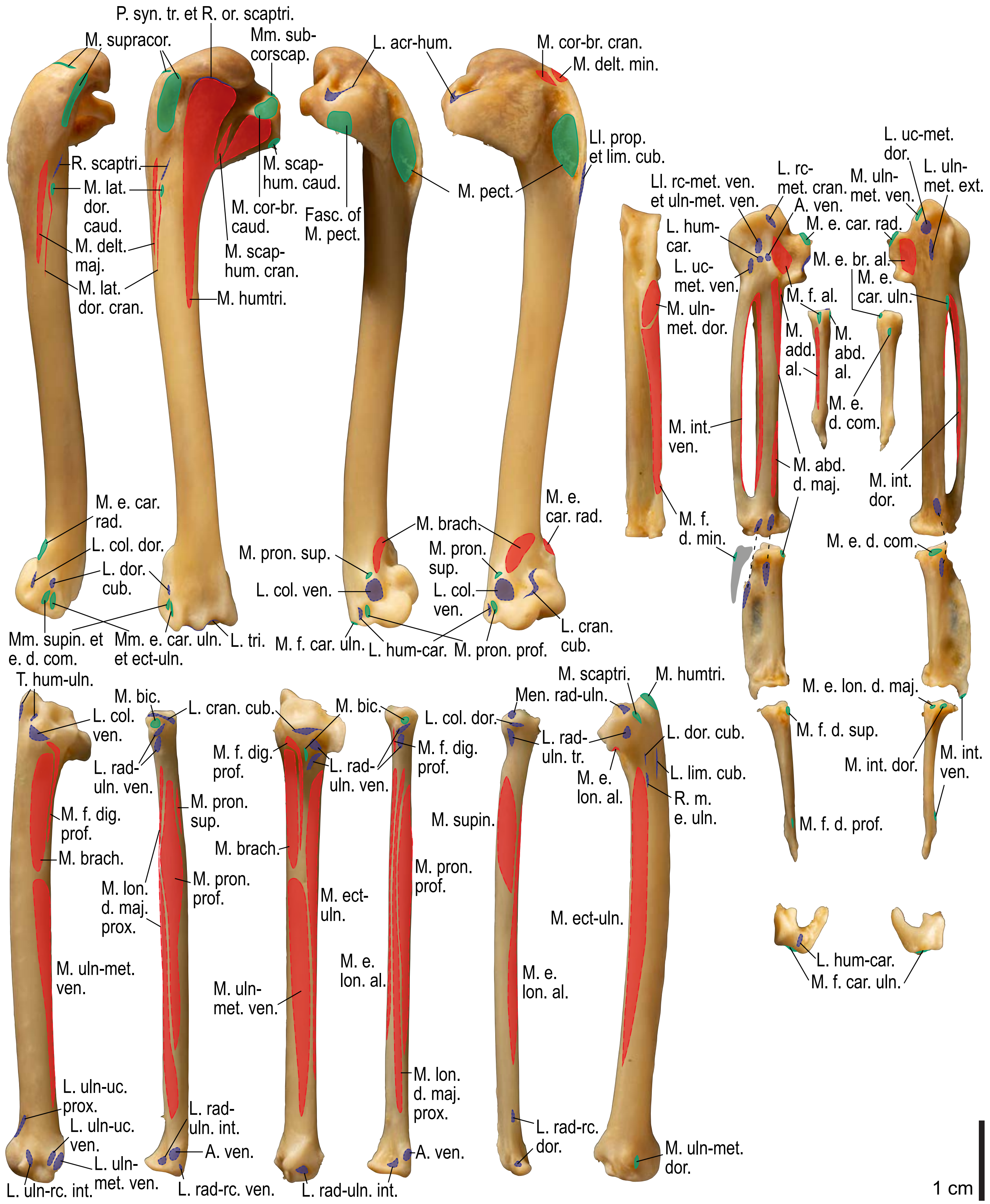

Figure S14. Osteological correlates of major wing muscles and ligaments in the wing of *Cepphus carbo*. Drawn on KUGM RAJ AO13062101. See Figures S2, S4, and S13 for legends.

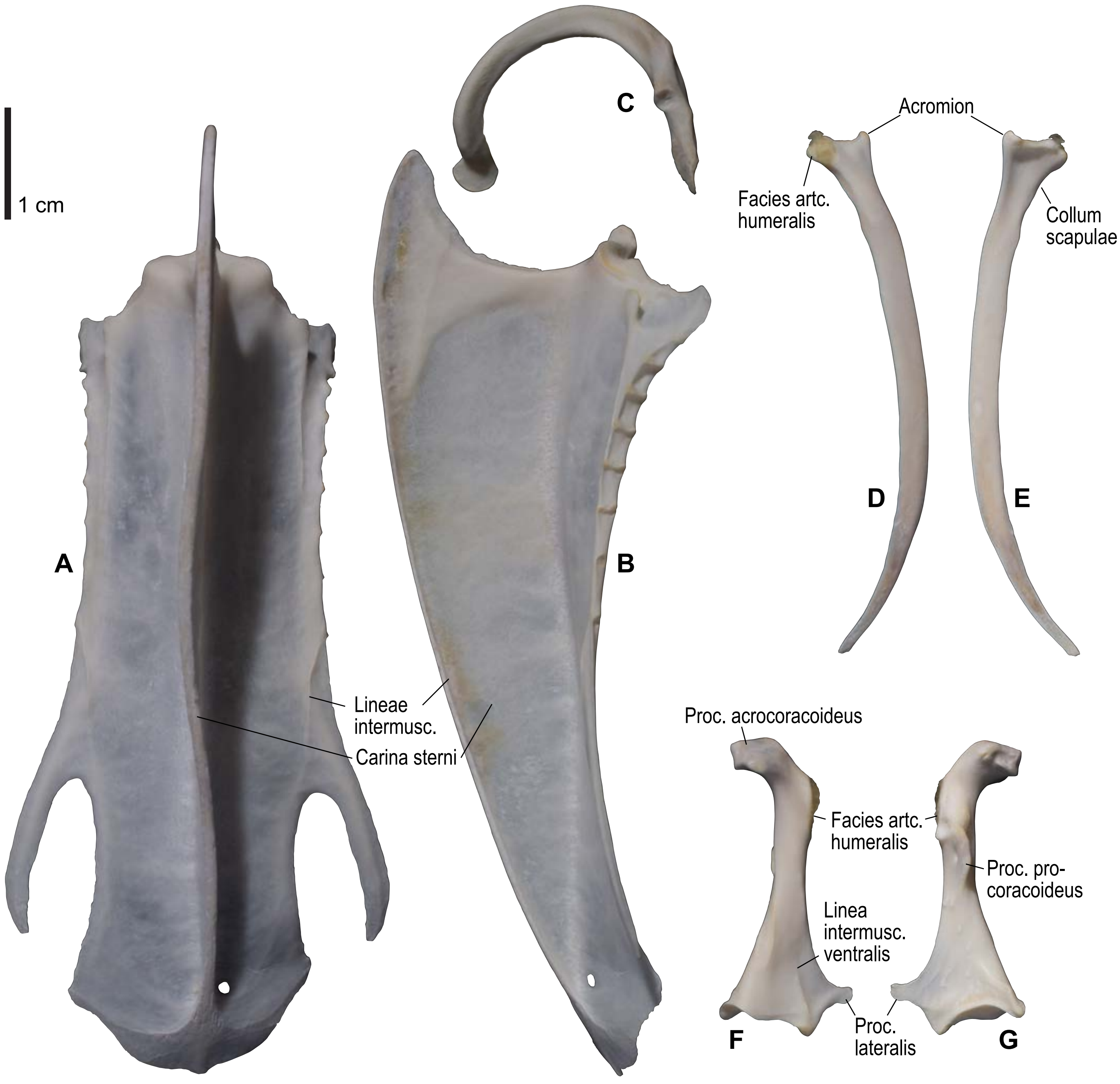

Figure S15. Osteology of the pectoral girdle of *Synthliboramphus antiquus*. Drawn on KUGM RA 1311. Sternum in ventral (**A**) and left lateral (**B**) views; furcula in left lateral view (**C**); left scapula in lateral (**D**) and medial (**E**) views; left coracoid in ventral (**F**) and dorsal (**G**) views. **B** and **C** are roughly aligned in their original relative positions and orientations. See Figure S1 for abbreviations.

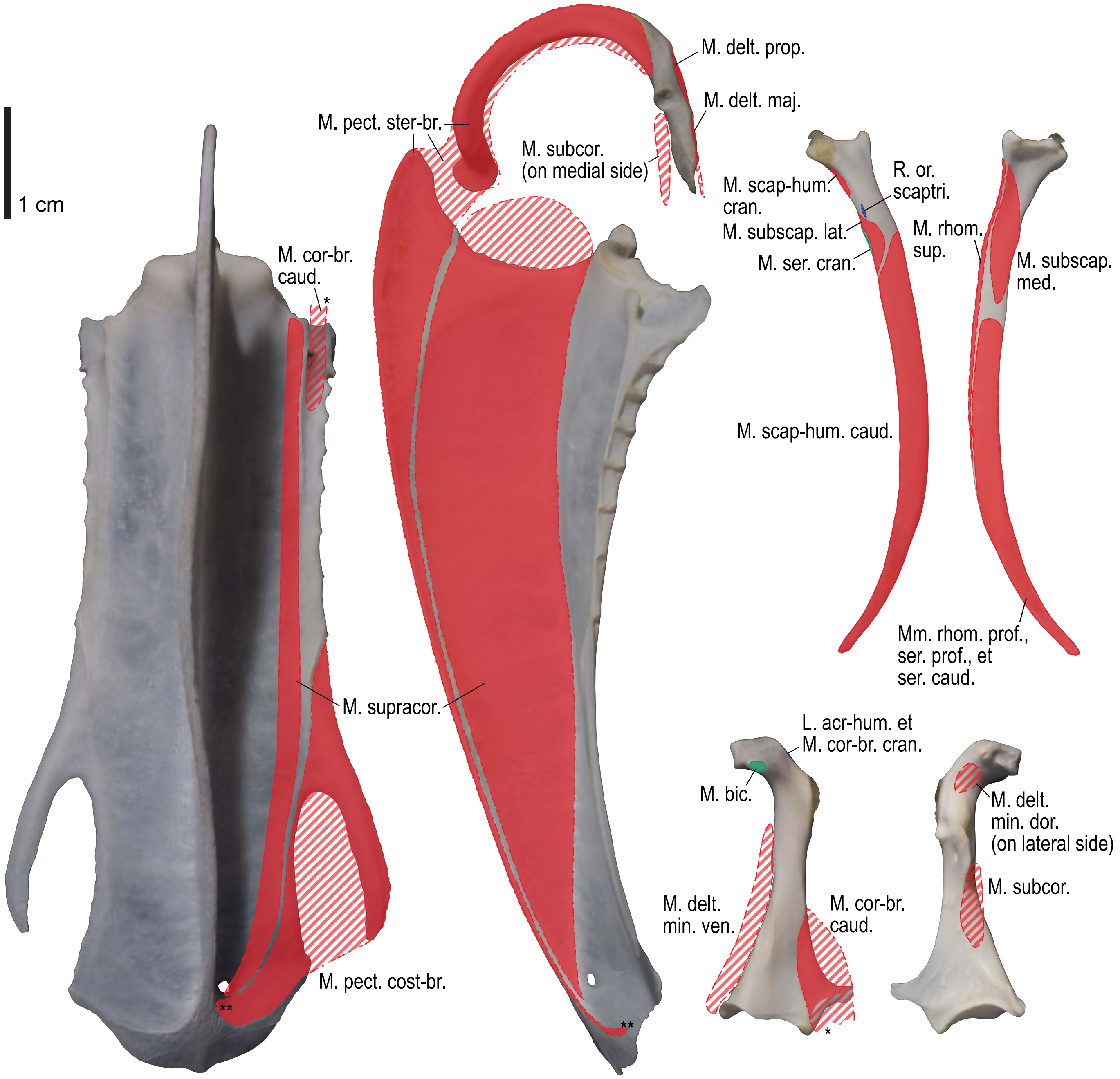

Figure S16. Osteological correlates of major wing muscles and ligaments in the pectoral girdle of *Synthliboramphus antiquus*. Drawn on KUGM RA 1311. See Figure S2 for legends.

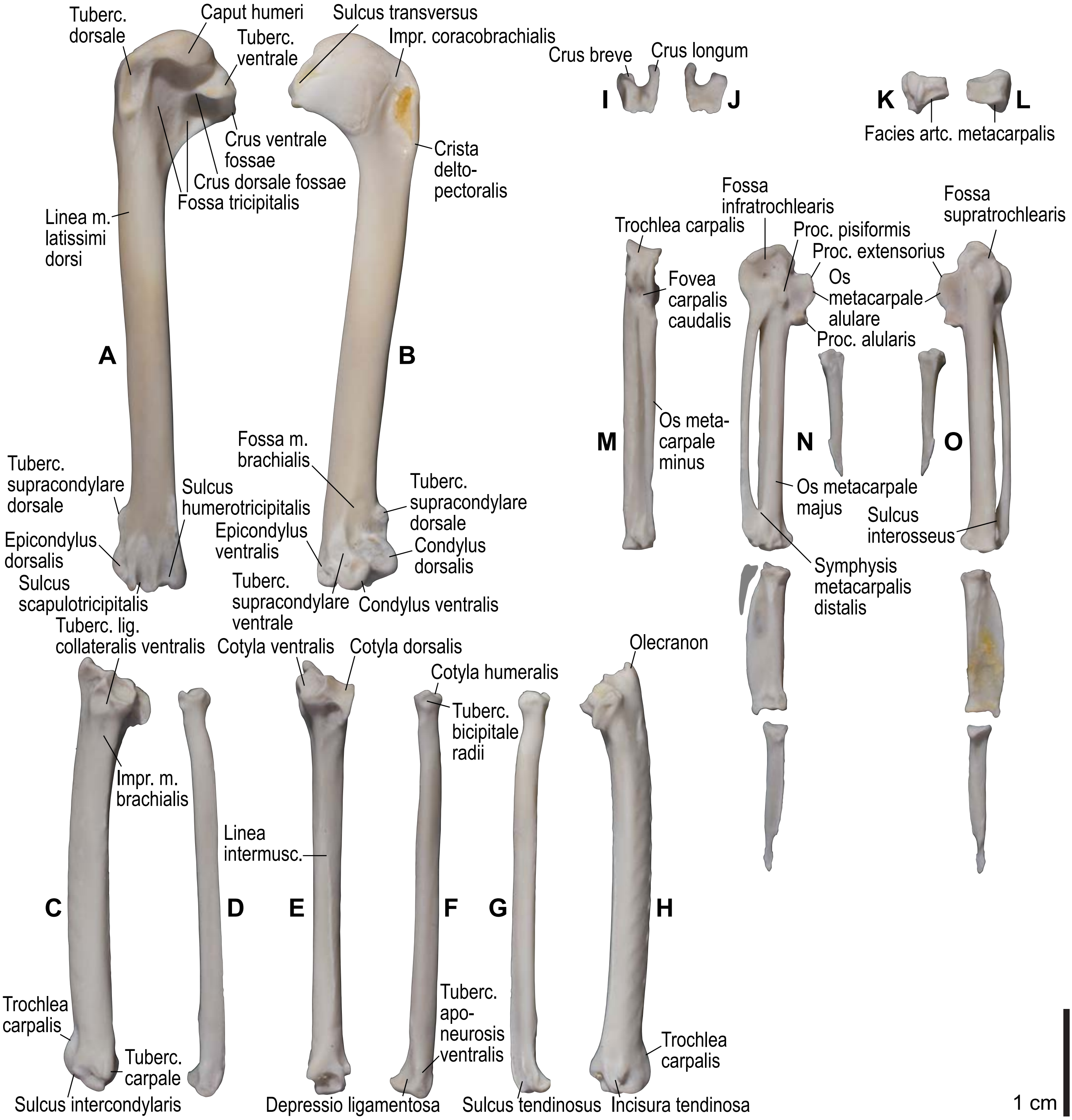

Figure S17. Osteology of the wing of *Synthliboramphus antiquus*. Drawn on KUGM RA 1311. Left humerus in caudal (**A**) and cranial (**B**) views; left ulna in ventral (**C**), cranial (**E**), and dorsal (**H**) views; left radius in ventral (**D**), caudoventral (**F**), and dorsal (**G**) views; left ulnare in proximal (**I**) and distal (**J**) views; left radiale in cranial (**K**) and caudal (**L**) views; left carpometacarpus and phalanges in caudal (**M**; phalanges not shown), ventral (**N**), and dorsal (**O**) views. Approximate outline of the missing phalanx of the minor digit is shown with gray shading. See Figure S1 for abbreviations.

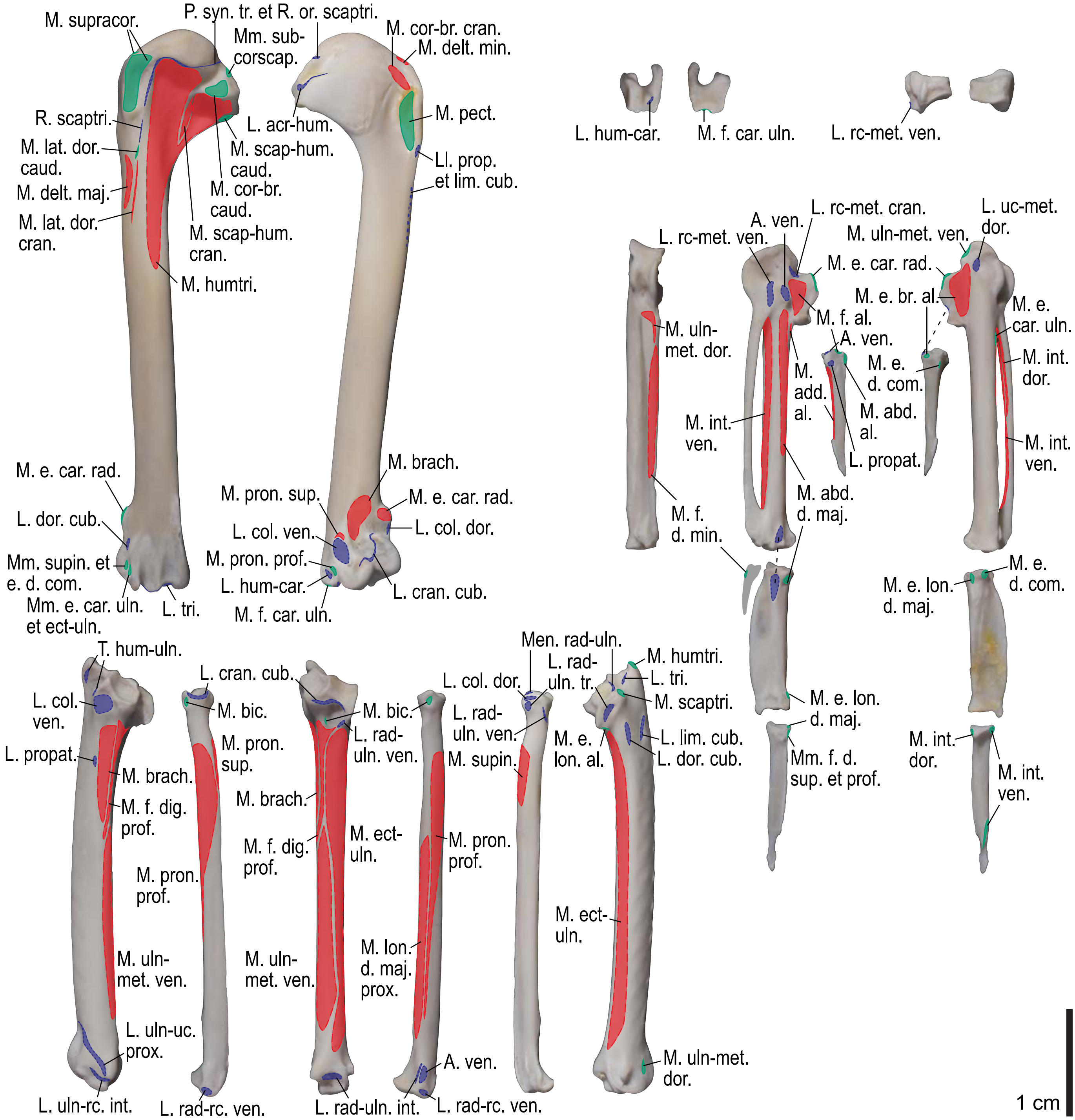

Figure S18. Osteological correlates of major wing muscles and ligaments in the wing of *Synthliboramphus antiquus*. Drawn on KUGM RA 1311. See Figures S2, S4, and S17 for legends.

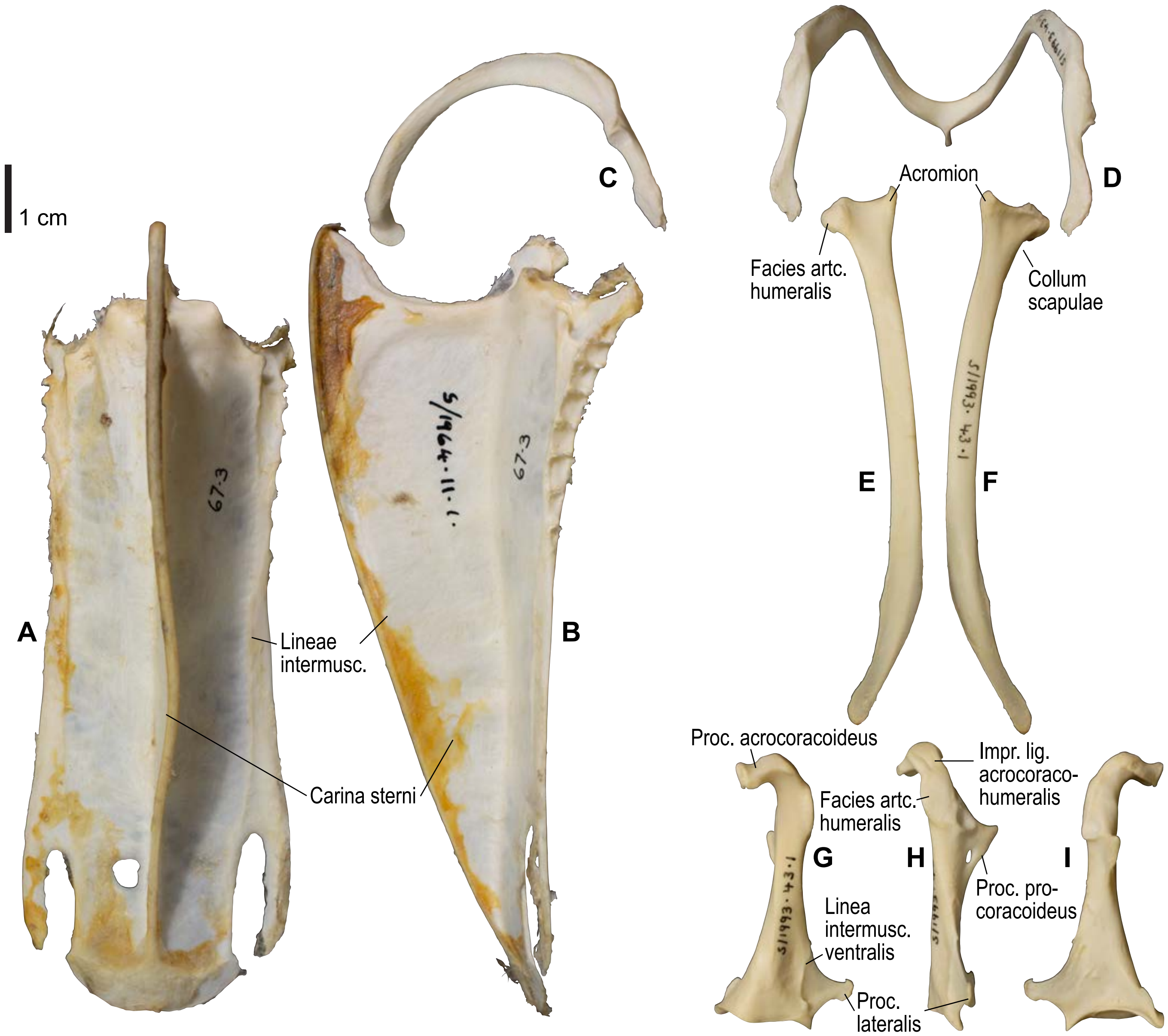

Figure S19. Osteology of the pectoral girdle of *Uria lomvia*. Drawn on NHMUK S/1964.11.1 (sternum) and S/1993.43.1 (other elements). Sternum in ventral (**A**) and left lateral (**B**) views; furcula in left lateral (**C**) and dorsal (**D**) views; left scapula in lateral (**E**) and medial (**F**) views; left coracoid in ventral (**G**), lateral (**H**) and dorsal (**I**) views. **B** and **C** are roughly aligned in their original relative positions and orientations. See Figure S1 for abbreviations.

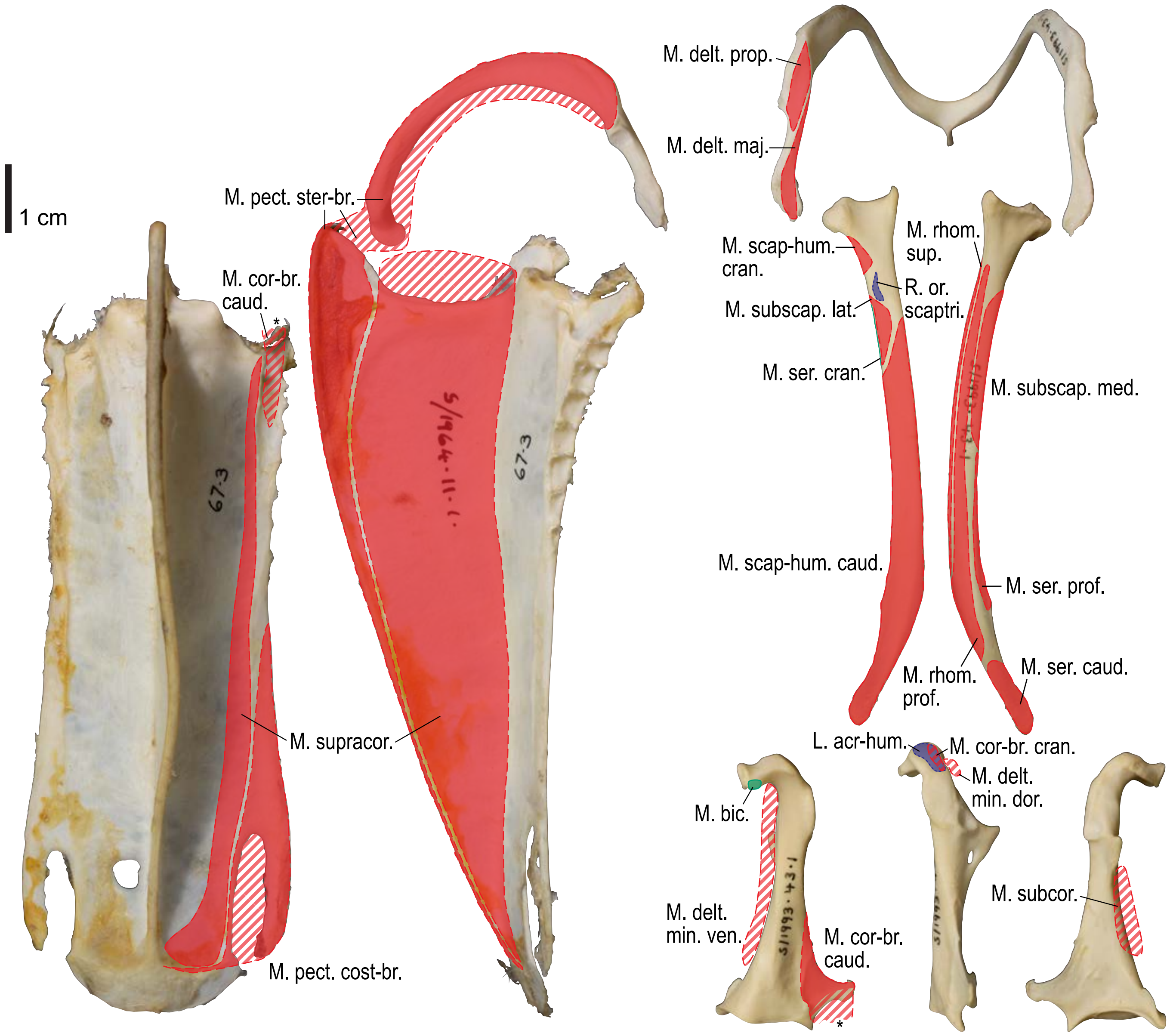

Figure S20. Osteological correlates of major wing muscles and ligaments in the pectoral girdle of *Uria lomvia*. Drawn on NHMUK S/1964.11.1 (sternum) and S/1993.43.1 (other elements). See Figure S2 for legends.

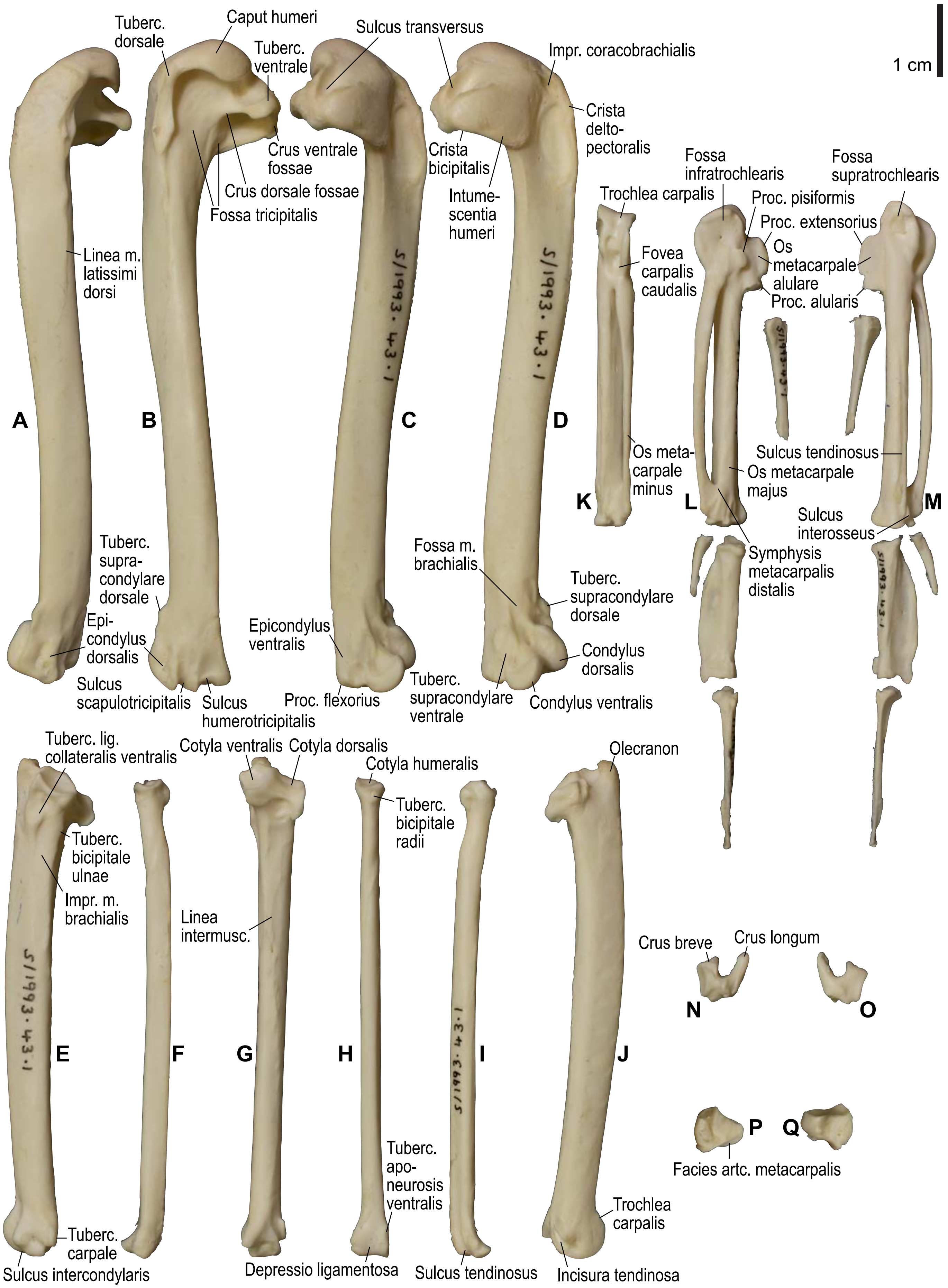

Figure S21. Osteology of the wing of *Uria lomvia*. Drawn on NHMUK S/1993.43.1. Left humerus in dorsal (**A**), caudal (**B**), ventral (**C**), and cranial (**D**) views; left ulna in ventral (**E**), cranial (**G**), and dorsal (**J**) views; left radius in ventral (**F**), caudal (**H**), and dorsal (**I**) views; left carpometacarpus and phalanges in caudal (**K**; phalanges not shown), ventral (**L**), and dorsal (**M**) views; left ulnare in proximal (**N**) and distal (**O**) views; right radiale (mirrored for comparison) in cranial (**P**) and caudal (**Q**) views. See Figure S1 for abbreviations.

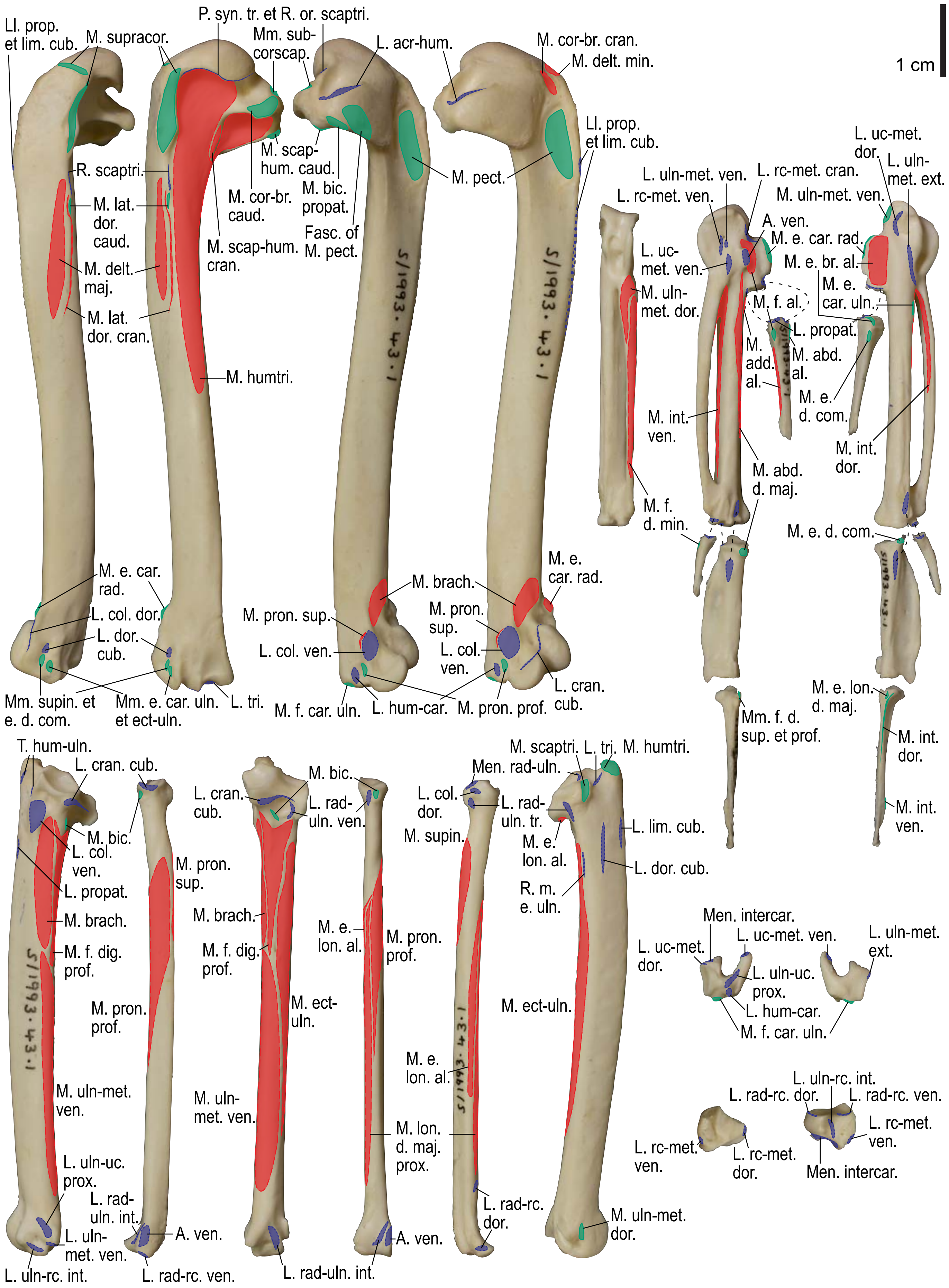

Figure S22. Osteological correlates of major wing muscles and ligaments in the wing of *Uria lomvia*. Drawn on NHMUK S/1993.43.1. See Figures S2 and S4 for legends.

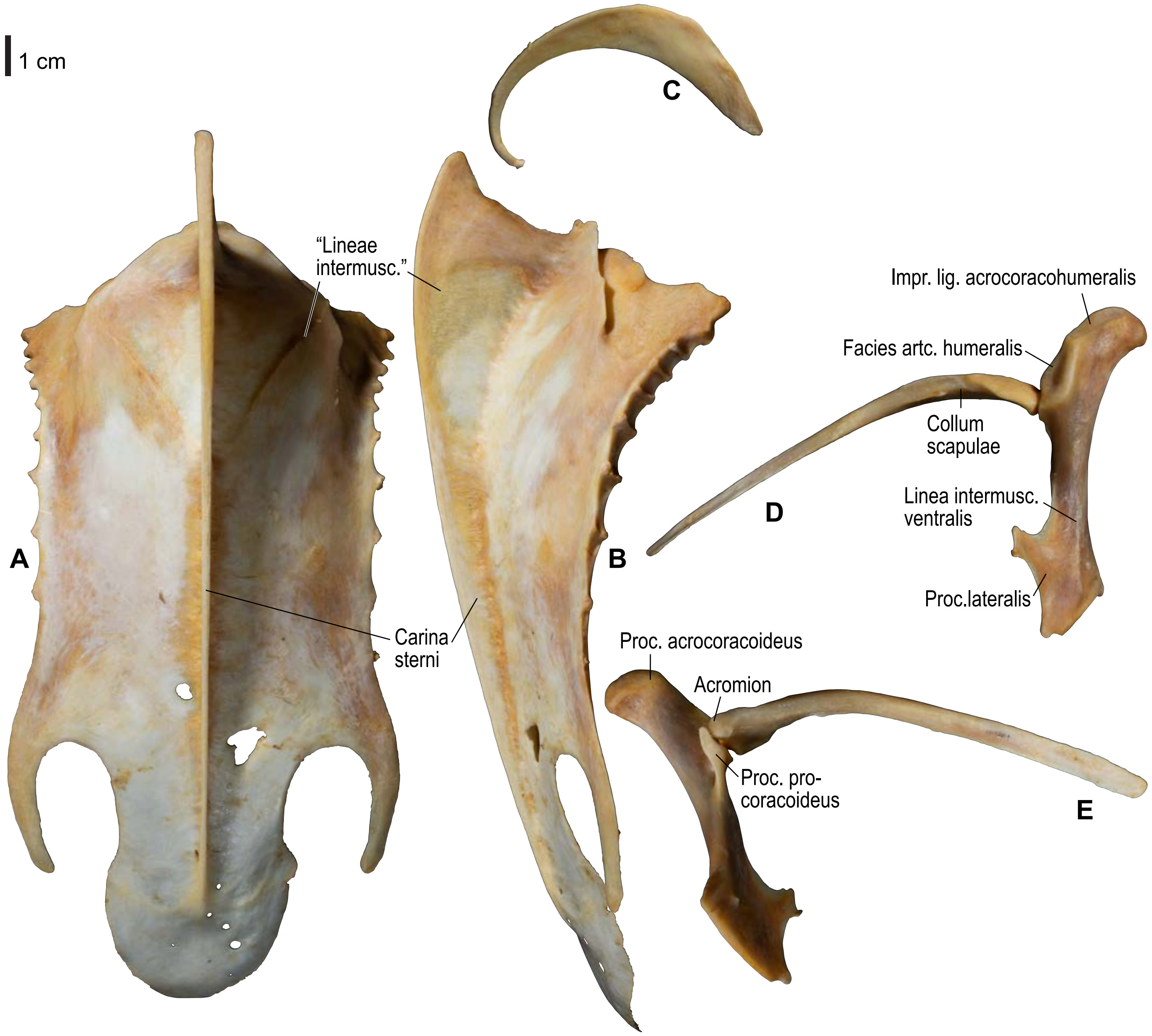

Figure S23. Osteology of the pectoral girdle of *Gavia adamsii*. Drawn on KUGM RAJ AO14052401. Sternum in ventral (**A**) and left lateral (**B**) views; furcula in left lateral view (**C**); right scapula and coracoid in lateral (**D**) and medial (**E**) views. **B** and **C** are roughly aligned in their original relative positions and orientations. See Figure S1 for abbreviations.

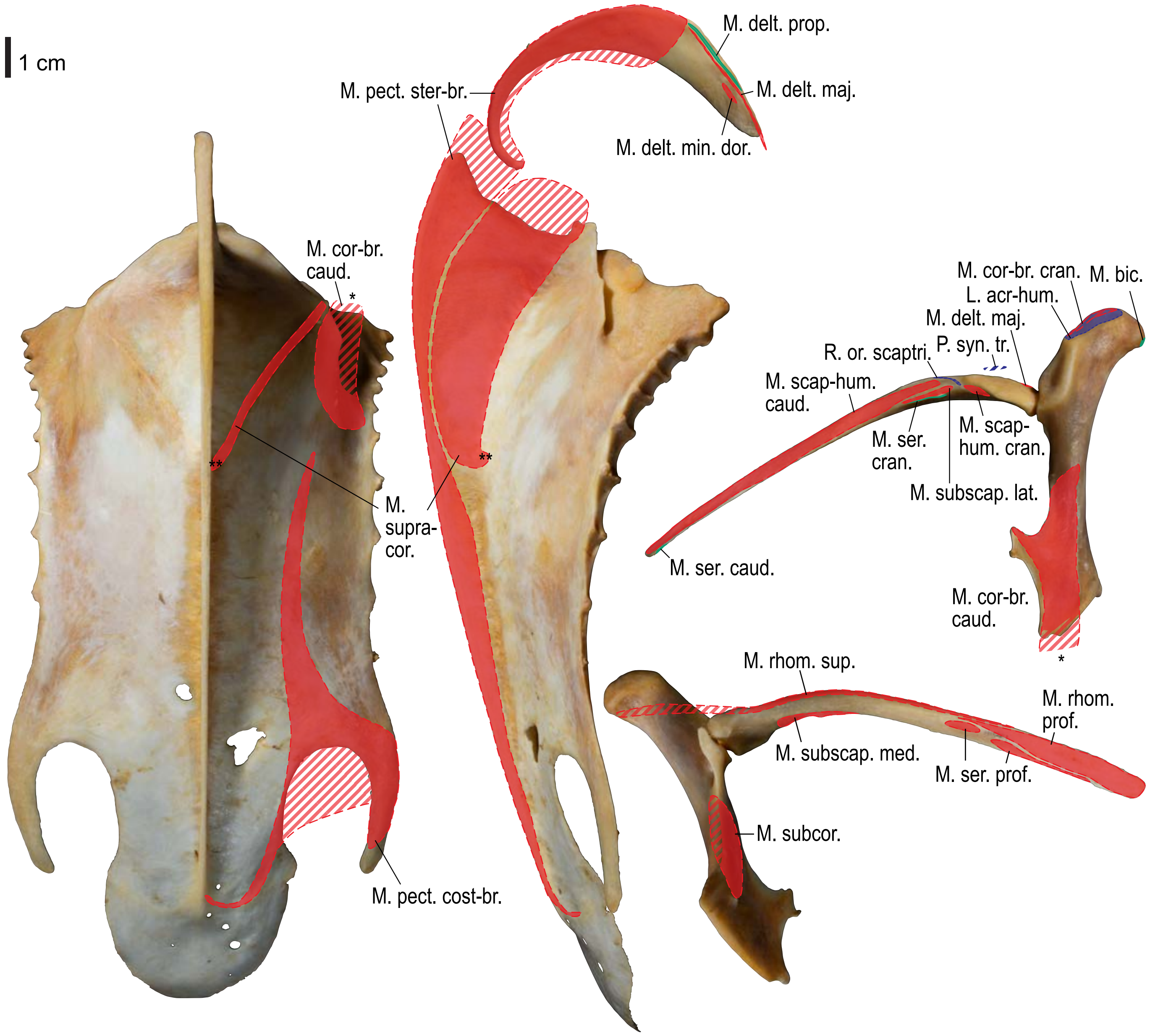

Figure S24. Osteological correlates of major wing muscles and ligaments in the pectoral girdle of *Gavia adamsii*. Drawn on KUGM RAJ AO14052401. See Figure S2 for legends.

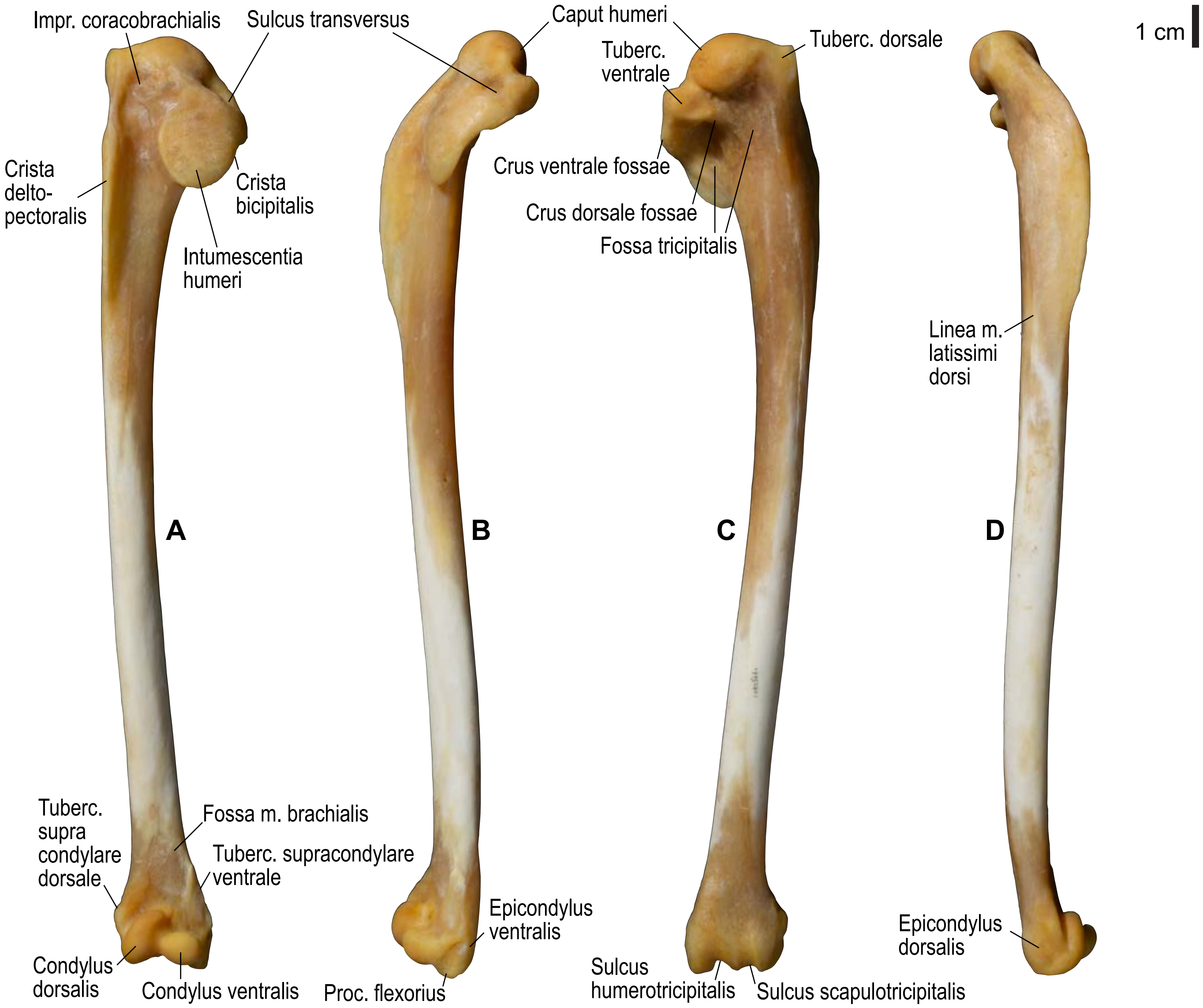

Figure S25. Osteology of the humerus of *Gavia adamsii*. Drawn on KUGM RAJ AO14052401. Right humerus in cranial (**A**), ventral (**B**), caudal (**C**), and dorsal (**D**) views. See Figure S1 for abbreviations.

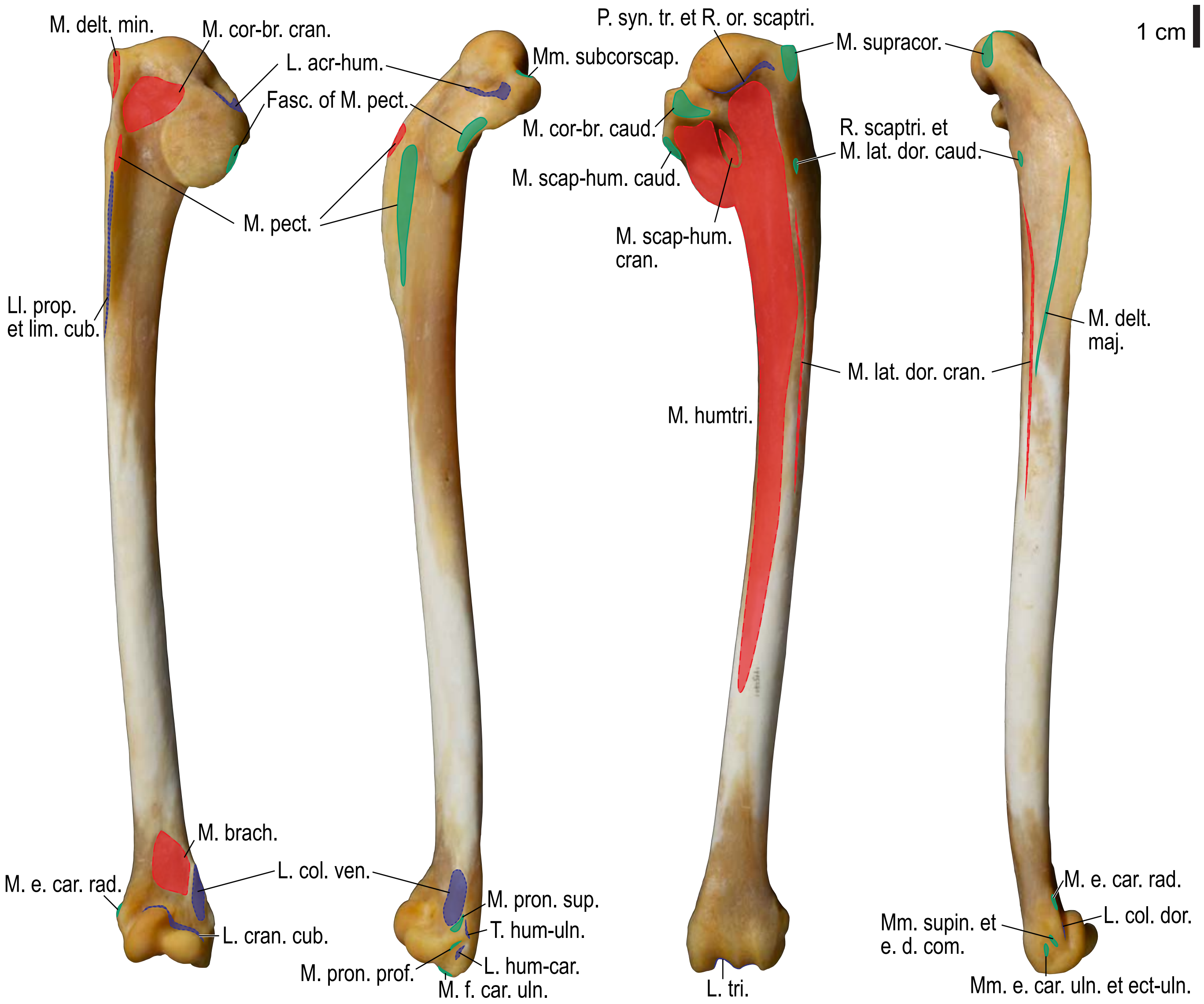

Figure S26. Osteological correlates of major wing muscles and ligaments in the humerus of *Gavia adamsii*. Drawn on KUGM RAJ AO14052401. See Figure S2 for legends.

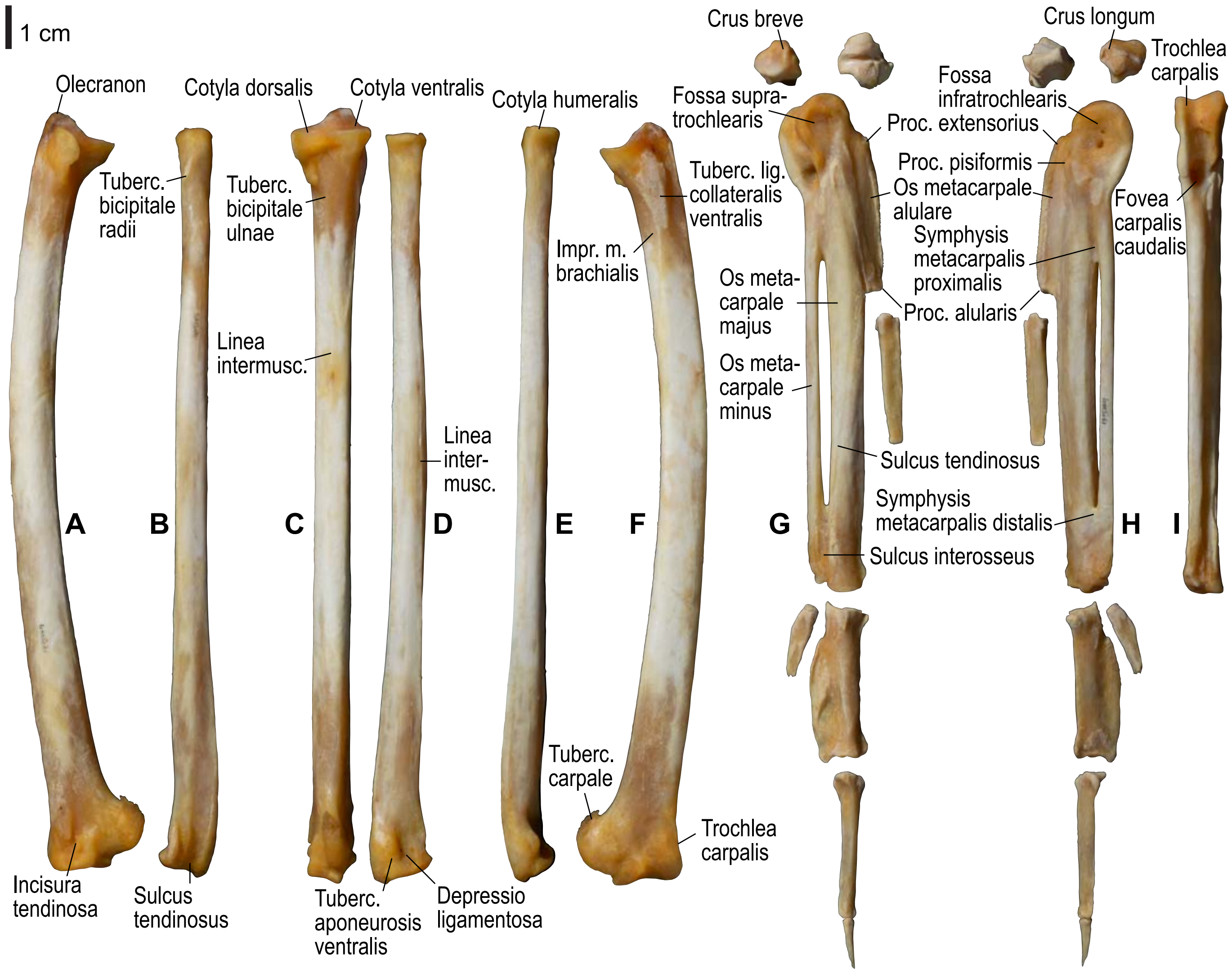

Figure S27. Osteology of the distal wing of *Gavia adamsii*. Drawn on KUGM RAJ AO14052401. Right ulna in dorsal (**A**), cranial (**C**), and ventral (**F**) views; right radius in dorsal (**B**), caudal (**D**), and ventral (**E**) views; right ulnare, radiale, carpometacarpus, and phalanges in dorsal (**G**), ventral (**H**), and caudal (**I**; carpometacarpus only) views. See Figure S1 for abbreviations.

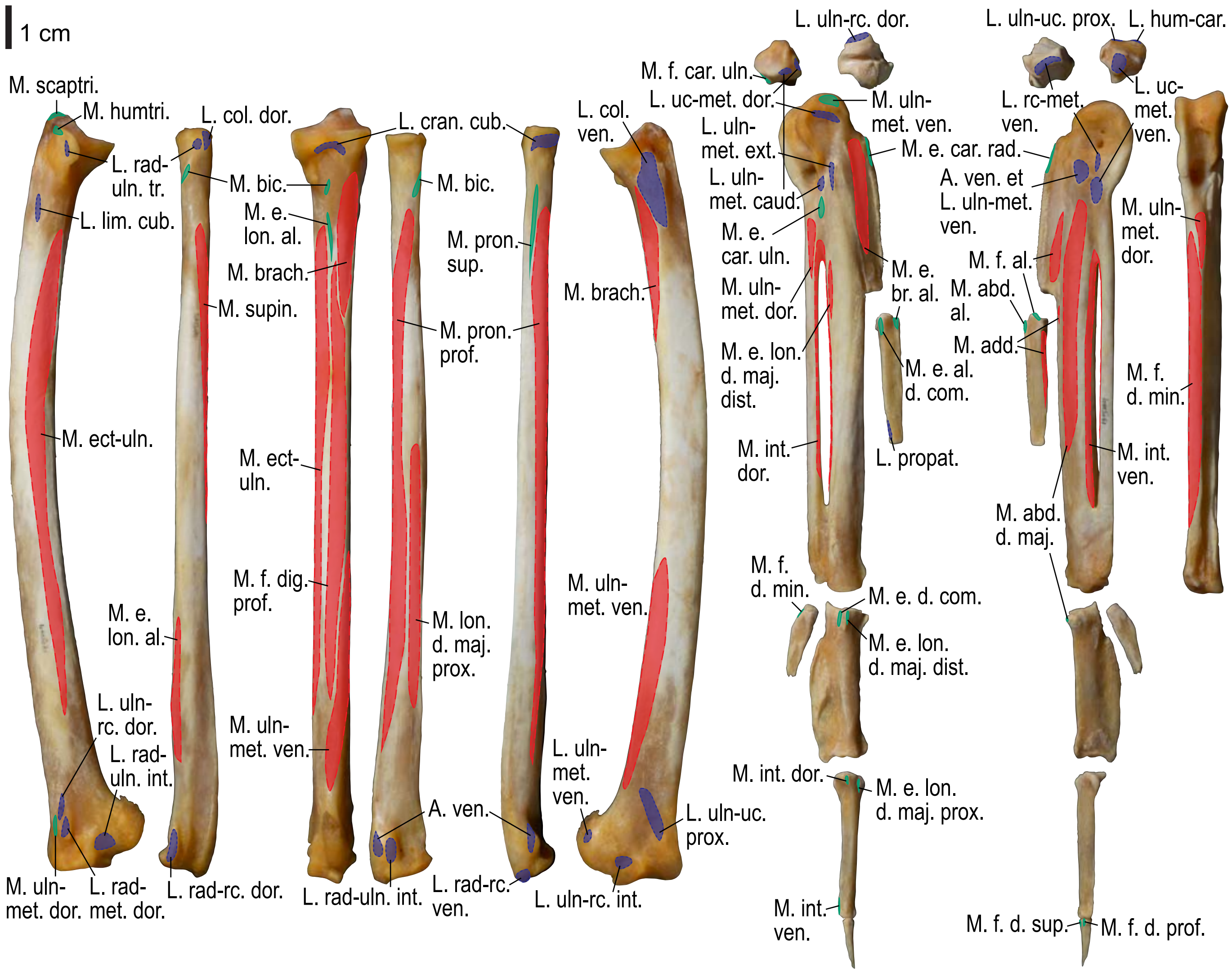

Figure S28. Osteological correlates of major wing muscles and ligaments in the distal wing of *Gavia adamsii*. Drawn on KUGM RAJ AO14052401. See Figure S2 for legends.

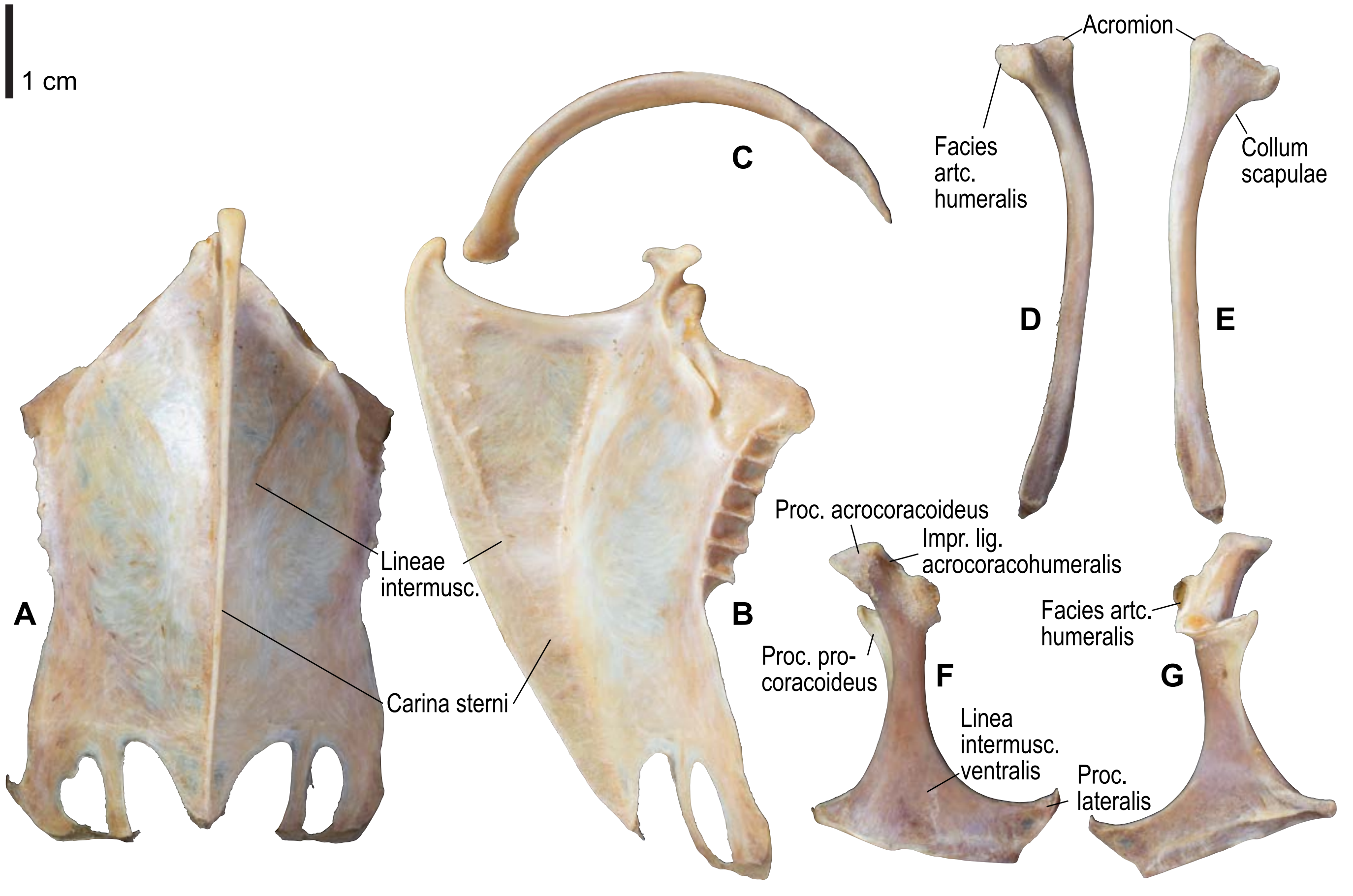

Figure S29. Osteology of the pectoral girdle of *Ardenna tenuirostris*. Drawn on KUGM RAJ AO09110484. Sternum in ventral (**A**) and left lateral (**B**) views; furcula in left lateral view (**C**); left scapula in lateral (**D**) and medial (**E**) views; left coracoid in ventral (**F**) and dorsal (**G**) views. **B** and **C** are roughly aligned in their original relative positions and orientations. See Figure S1 for abbreviations.

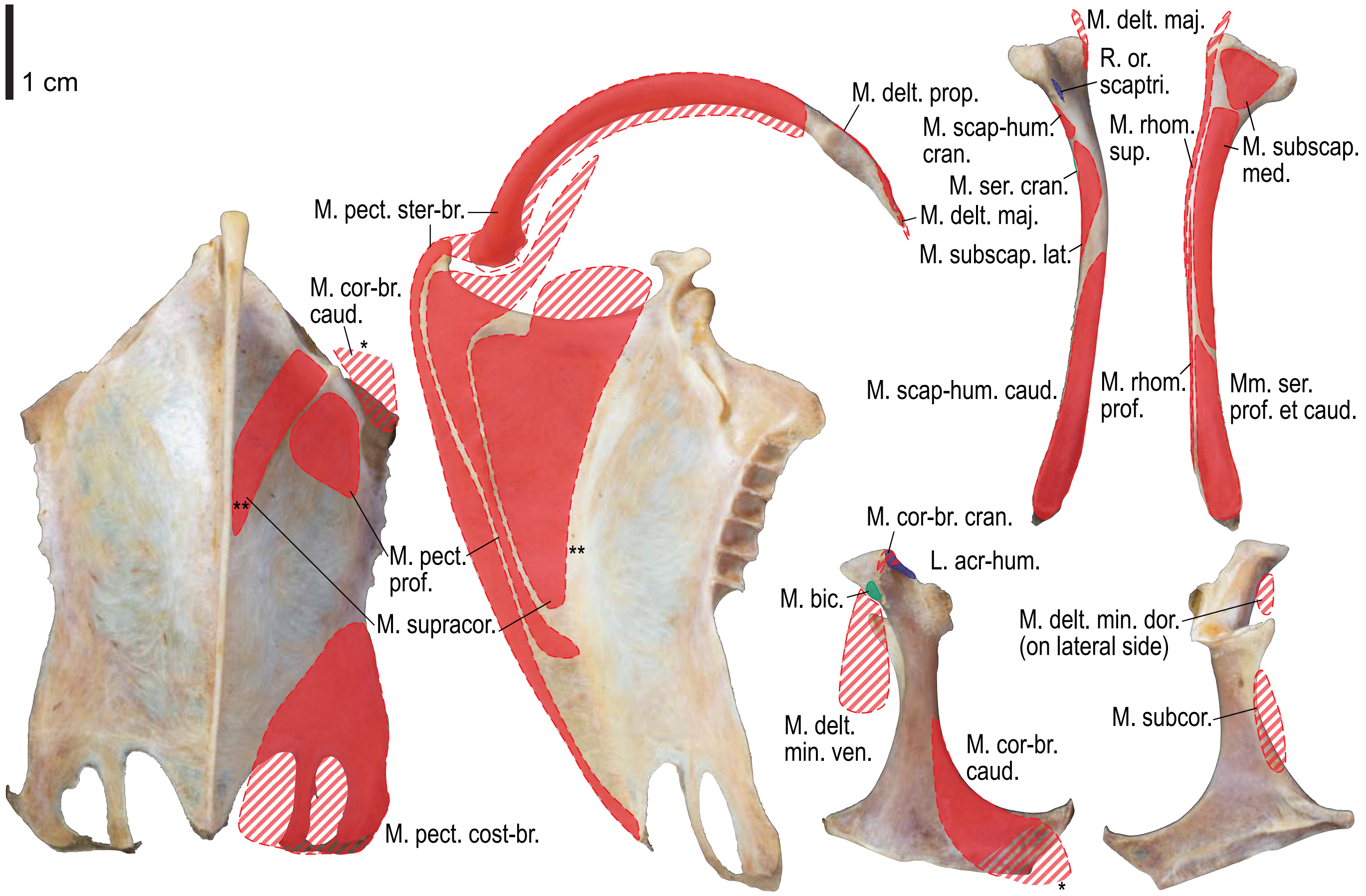

Figure S30. Osteological correlates of major wing muscles and ligaments in the pectoral girdle of *Ardenna tenuirostris*. Drawn on KUGM RAJ AO09110484. See Figure S2 for legends.

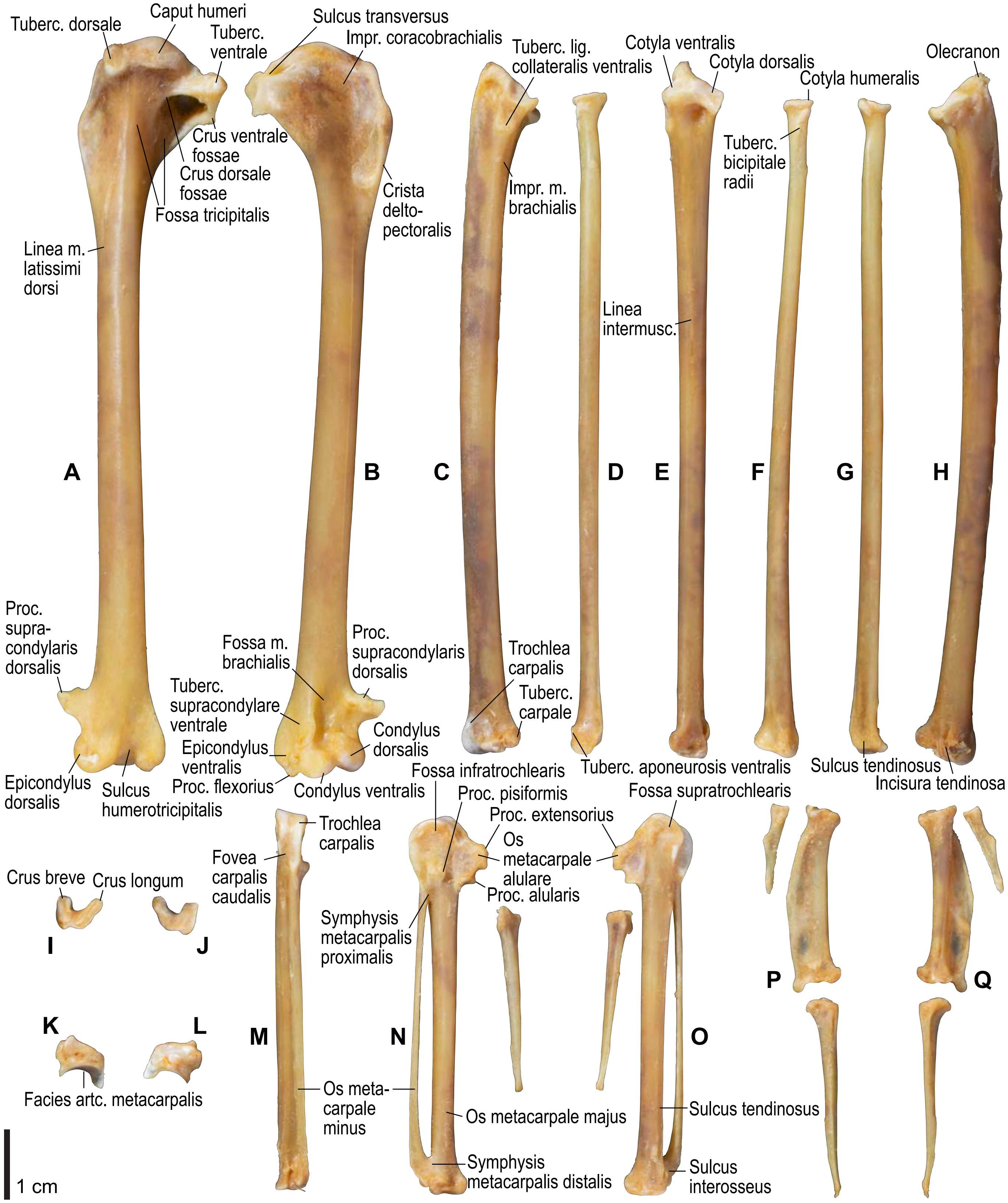

Figure S31. Osteology of the wing of *Ardenna tenuirostris*. Drawn on KUGM RAJ AO09110484. Left humerus in caudal (**A**) and cranial (**B**) views; left ulna in ventral (**C**), cranial (**E**), and dorsal (**H**) views; left radius in ventral (**D**), caudal (**F**), and dorsal (**G**) views; left ulnare in proximal (**I**) and distal (**J**) views; left radiale in cranial (**K**) and caudal (**L**) views; left carpometacarpus and phalanges in caudal (**M**; phalanges not shown), ventral (**N**, **P**), and dorsal (**O**, **Q**) views. See Figure S1 for abbreviations.

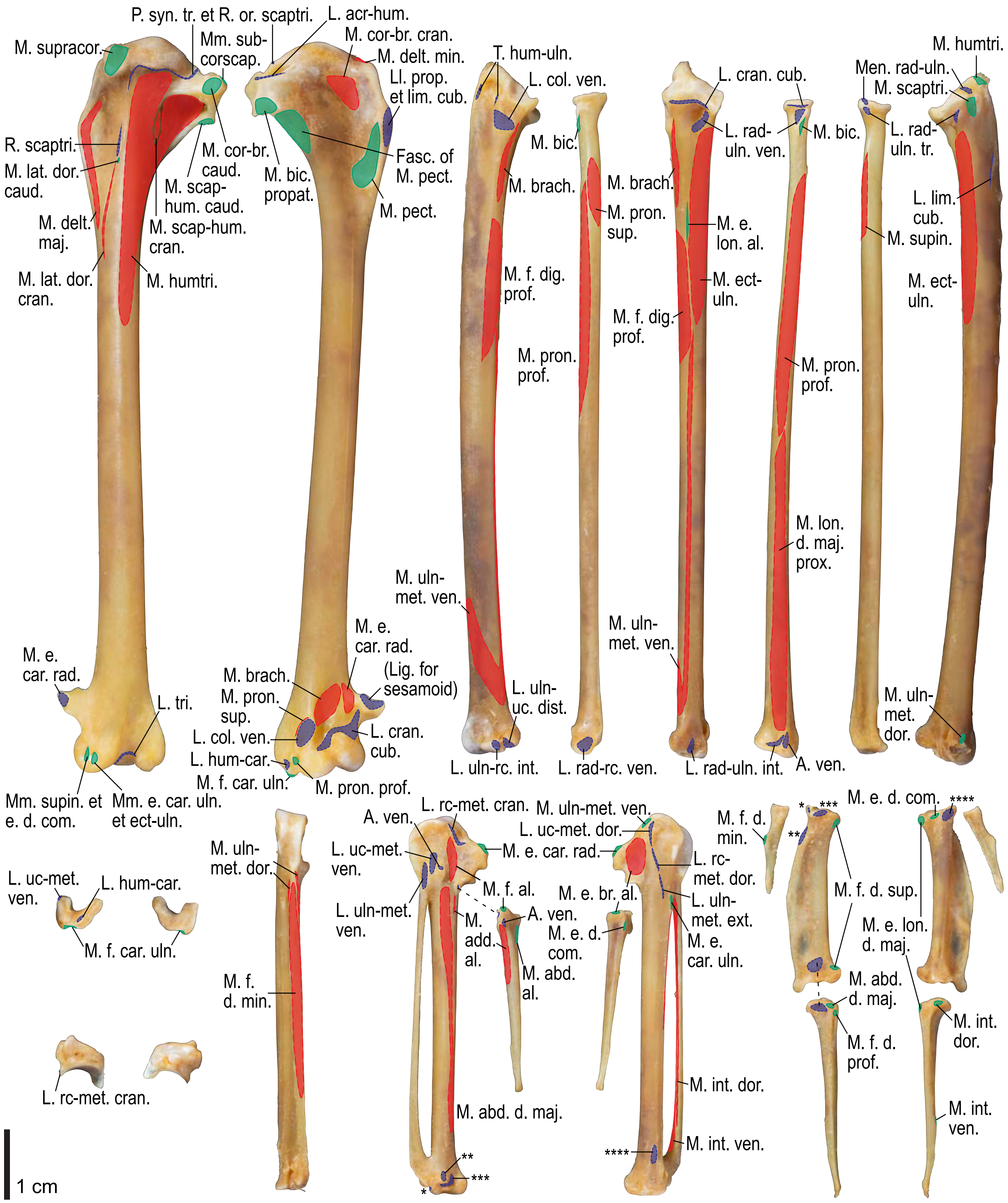

Figure S32. Osteological correlates of major wing muscles and ligaments in the wing of *Ardenna tenuirostris*. Drawn on KUGM RAJ AO09110484. See Figures S2 and S4 for legends. Correspondence of attachment sites of some ligaments across panels are shown with asterisks.
